# From Abstract Symbols to Emotional (In-)Sights: An Eye Tracking Study on the Effects of Emotional Vignettes and Pictures

**DOI:** 10.3389/fpsyg.2020.00905

**Published:** 2020-05-26

**Authors:** Franziska Usée, Arthur M. Jacobs, Jana Lüdtke

**Affiliations:** ^1^Department of Experimental and Neurocognitive Psychology, Freie Universität Berlin, Berlin, Germany; ^2^Center for Cognitive Neuroscience Berlin, Freie Universität Berlin, Berlin, Germany

**Keywords:** reading, vignettes, pictures, emotion induction, ratings, valence, eye movements

## Abstract

Reading is known to be a highly complex, emotion-inducing process, usually involving connected and cohesive sequences of sentences and paragraphs. However, most empirical results, especially from studies using eye tracking, are either restricted to simple linguistic materials (e.g., isolated words, single sentences) or disregard valence-driven effects. The present study addressed the need for ecologically valid stimuli by examining the emotion potential of and reading behavior in emotional vignettes, often used in applied psychological contexts and discourse comprehension. To allow for a cross-domain comparison in the area of emotion induction, negatively and positively valenced vignettes were constructed based on pre-selected emotional pictures from the *Nencki Affective Picture System* (NAPS; Marchewka et al., 2014). We collected ratings of perceived valence and arousal for both material groups and recorded eye movements of 42 participants during reading and picture viewing. Linear mixed-effects models were performed to analyze effects of valence (i.e., valence category, valence rating) and stimulus domain (i.e., textual, pictorial) on ratings of perceived valence and arousal, eye movements in reading, and eye movements in picture viewing. Results supported the success of our experimental manipulation: emotionally positive stimuli (i.e., vignettes, pictures) were perceived more positively and less arousing than emotionally negative ones. The cross-domain comparison indicated that vignettes are able to induce stronger valence effects than their pictorial counterparts, no differences between vignettes and pictures regarding effects on perceived arousal were found. Analyses of eye movements in reading replicated results from experiments using isolated words and sentences: perceived positive text valence attracted shorter reading times than perceived negative valence at both the supralexical and lexical level. In line with previous findings, no emotion effects on eye movements in picture viewing were found. This is the first eye tracking study reporting superior valence effects for vignettes compared to pictures and valence-specific effects on eye movements in reading at the supralexical level.

## Introduction

Imagine a future where the best-selling books aren’t the sole product of an author’s mind but the result of a machine learning assisted approach. A future with personalized phrases and e-books being able to predict your reading behavior. What would be the key to a future like this? Concerning psychological reading research, it would certainly require a stronger focus on ecologically valid study materials (Jacobs, 2015a; Pinheiro et al., 2017; Xue et al., 2019). In this context, most empirical results, especially from studies using eye tracking, are limited to the level of single words or experimentally controlled sentences (Clifton et al., 2007; Radach et al., 2008; Radach and Kennedy, 2013; Wallot et al., 2013). By contrast, reading as one of the essential daily activities commonly involves context information and goes along with emotional processes (e.g., Jacobs, 2011; Mar et al., 2011; Bohn-Gettler, 2019). This leads unavoidably to the second key point. The future scenario calls for a better understanding of affective responses elicited by ecologically valid text stimuli. In discourse comprehension, many studies made use of textual materials and indicated, for example, that the emotions of protagonists were represented in situation models even when not explicitly mentioned (e.g., Gernsbacher et al., 1992; Gygax et al., 2003, 2004, 2007). However, such studies largely neglected both reader’s emotions and valence-driven effects.

The scientific investigation of affective processes necessitates the availability of standardized stimuli that are reliably able to elicit emotions under controlled, experimental conditions. At present, researchers have access to a variety of cross-validated, international databases addressing different perceptual modalities and providing normative ratings. However, verbal stimulus sets are again restricted to the level of words (e.g., Bradley and Lang, 1999; Redondo et al., 2007; Võ et al., 2009; Eilola and Havelka, 2010; Briesemeister et al., 2011; Soares et al., 2012; Moors et al., 2013; Söderholm et al., 2013; Warriner et al., 2013; Montefinese et al., 2014; Schmidtke et al., 2014; Riegel et al., 2015; Imbir, 2016a) or single sentences (Bradley and Lang, 2007; Imbir, 2016b; Pinheiro et al., 2017). In addition, their use as an emotion induction method has been predominated by visual stimuli such as pictures (Dan-Glauser and Scherer, 2011). However, only little attention has been paid to the comparison of verbal and visual stimulus domains. For example, early meta-analyses on the efficiency of emotion induction procedures neither differentiated between stories and films nor included static pictures (Gerrards-Hesse et al., 1994; Westermann et al., 1996). Even when differentiated and included, the heterogenous definition of vignettes (cf. Siedlecka and Denson, 2019) made it difficult to draw conclusions about their suitability.

With respect to simpler linguistic materials, Schlochtermeier et al. (2013) were able to highlight potentially beneficial effects of words and phrases on evaluative judgments. More specifically, their behavioral results revealed stronger valence ratings for verbal compared to pictorial stimuli. Additionally, no differences in reaction times and arousal ratings were reported contradicting the commonly assumed privileged processing of pictures within the area of emotion induction (e.g., Azizian et al., 2006; Kensinger and Schacter, 2006; Seifert, 1997). Similarly, Bayer and Schacht (2014) were able to show that both words, faces, and pictures elicit early and late emotion effects as indicated by event-related potentials. Furthermore, words and pictorial materials were perceived as comparably strong in their emotional valence and arousal.

The present study was designed to face some of the aforementioned challenges by introducing a set of ecologically valid, emotion-inducing vignettes verbalizing the semantic content of pre-selected pictures from the *Nencki Affective Picture System* (NAPS; Marchewka et al., 2014). For both textual and pictorial stimuli, eye movements, as sensitive measure for cognitive and affective processes (Rayner, 1998, 2009), were recorded and analyzed. Accordingly, our study aimed at extending prior findings with two main objectives: (1) the comparison between more complex verbal (i.e., textual) and visual affective materials in the area of emotion induction, and (2) the influence of emotional content on reading behavior in ecologically valid texts.

The article is organized as follows. After reviewing past research on emotion in reading and picture viewing, effects of Valence Category (i.e., positive, negative) and Stimulus Domain (i.e., textual, pictorial) on ratings of Perceived Valence and Arousal are analyzed. Second, the influence of (perceived) textual valence on reading times of both supralexical (i.e., text level) and lexical units (i.e., word level) is examined. Lastly, the role of pictorial valence on the execution of fixations (i.e., Mean Fixation Duration, Total Number of Fixations) and saccades (i.e., Mean Saccade Amplitude) is illustrated. While eye tracking data are reported for both stimulus domains, the thematic focus and innovation of the present article strongly lies on the emotional processes evoked by linguistic stimuli.

## Emotion in Reading and Picture Viewing

### Emotion in Reading

The expression of emotion belongs to the crucial functions of human language (Bühler, 1934; Koelsch et al., 2015). However, for a long time affective processes during reading have only played a minor role in empirical research (Jacobs, 2011). Nowadays, there is empirical evidence supporting the behavioral and neuronal effects of the emotion potential of both simple and complex linguistic materials (e.g., Citron, 2012; Hsu et al., 2014, 2015a,b,c; Jacobs et al., 2015, 2016a; Lüdtke and Jacobs, 2015). More precisely, differences in the processing of neutral and emotional verbal stimuli are emphasized at the lexical (Kuchinke et al., 2005; Kissler et al., 2006; Herbert et al., 2008; Hofmann et al., 2009; Kousta et al., 2009; Kissler and Herbert, 2013; Recio et al., 2014), sentential (Jiang et al., 2014; Ding et al., 2015; Knickerbocker et al., 2015; Lüdtke and Jacobs, 2015), and text levels (Altmann et al., 2012, 2014; Hsu et al., 2015a,b,c; Lehne et al., 2015; Ballenghein et al., 2019). According to Lüdtke and Jacobs (2015), emotional words are characterized by their affective meaning or explicit expression of emotion. In comparison to their neutral counterparts, they are assumed to possess privileged access to attentional and cognitive resources (e.g., Kuchinke et al., 2005; Kousta et al., 2009; Ding et al., 2015).

Supporting evidence for this was first provided by behavioral studies and experiments using EEG and fMRI (Citron, 2012, for review). Interestingly, also eye movement studies indicated differences in the processing of emotional compared to neutral words (Scott et al., 2012; Knickerbocker et al., 2015). These studies examined emotionally valenced (i.e., positive, negative) and neutral target words embedded in a single sentence structure. Word frequency was considered as additional manipulation by Scott et al. (2012). In both experiments, early measures of processing (e.g., single and first fixation duration) indicated faster reading of emotional words compared to neutral ones. Moreover, emotional valence seemed to be of similar advantage at later processing stages as reflected in shorter total reading times, less regressions, and shorter second pass reading (Knickerbocker et al., 2015). However, valence-specific effects remained unexplored in this comparison. Both studies replicated results from EEG and fMRI studies indicating that emotional words are easier to process than their neutral counterparts while highlighting some differences when comparing emotionally positive and negative words. In this context, modulatory effects of word frequency were reported (Scott et al., 2012). More specifically, negative valence was only found to be beneficial when targets were characterized by a low frequency. In contrast, processing advantages of emotionally positive words emerged robustly under all experimental conditions.

Following a dimensional approach of emotion, words’ emotion potential can be empirically and computationally quantified in a two-dimensional space with valence representing their polarity and arousal their intensity (Võ et al., 2006, 2009; Scott et al., 2012; Recio et al., 2014; Jacobs, 2019). Since the two variables are strongly intercorrelated, high arousal commonly goes in line with extreme valence (Bradley and Lang, 1994; Lang et al., 2008; Hofmann et al., 2009; Citron, 2012; Jacobs et al., 2015). Moreover, emotionally negative words tend to reach higher values than emotionally positive ones (e.g., Võ et al., 2009). Concerning valence-specific effects, positive events (e.g., words, sentences) are often associated with accelerated reactions and facilitated word processing (Kousta et al., 2009; Briesemeister et al., 2011; Lüdtke and Jacobs, 2015). In case of negative valence, the oftentimes inconclusive effects are mainly explained by the interactive relationship with the dimension of arousal. Thus, emotionally negative words are mainly associated with shorter reaction times when having high arousal values (Larsen et al., 2008; Hofmann et al., 2009; Recio et al., 2014). In sum, the current evidence supports the notion of superior processing of emotionally positive and high-arousal negative words.

Can we expect similar results when manipulating valence at the supralexical, textual level? According to Bestgen (1994), we can act on the assumption that there is a high correlation between the different processing levels. By collecting valence ratings of four texts and their constituting sentences and words, significant correlations between the three processing levels were shown. Similarly, Whissell (2003) demonstrated that valence and arousal ratings of words from the *Dictionary of Affect in Language* (Whissell and Dewson, 1986) can be used as an estimator of the affective tone of excerpts of romantic poetry. Finally, Hsu et al. (2015b) computed mean and spread measures of valence and arousal for the words of 120 text passages from the Harry Potter novels. Their results indicated that mean lexical valence values can account for approximately 28% of the variance in subjective valence ratings of the text units. Taken together, previous results suggest that the valence of supralexical units like the present vignettes can be – in its simplest form – predicted (at least approximately, cf. Lüdtke and Jacobs, 2015) as a function of the valence of their constituting words (Jacobs, 2015b).

#### Vignettes as Controlled More Natural Reading Material

As already pointed out, a majority of reading studies fails to go beyond the level of single words or non-literary constructed sentences, i.e., so-called textoids (Bailey and Zacks, 2011). Although this experimental approach offers the possibility to test specific assumptions, the results can only be generalized to a limited extent (Clifton and Staub, 2011). Frazier and Rayner (1982) were already able to show that fixation times are influenced by phrase structure. The context in which a word is presented plays a similarly crucial role (Kuperman et al., 2010; Clifton and Staub, 2011; Wallot et al., 2013). Hence, the use of single words neglects the entangled effects of both syntactic and supralexical semantic features (Boston et al., 2008). To overcome such limitations, existing narratives became an attractive alternative. In this regard, short stories (e.g., Altmann et al., 2012, 2014) and fairy tales (Wallentin et al., 2011), poems (Lüdtke et al., 2014; Jacobs et al., 2016b; Xue et al., 2019), excerpts of books such as the Harry Potter novels (e.g., Hsu et al., 2014, 2015a,b, 2015c), “The House Of The Scorpion” (Wallot et al., 2013), “The Sandman” (Jacobs, 2015b; Lehne et al., 2015; Jacobs and Lüdtke, 2017), “One Boy’s Day” (Speer et al., 2009), “Dubliners” (Cupchik et al., 1998), “Hurricane Hazel” (Cupchik and Laszlo, 1994), and newspaper articles (Kennedy and Pynte, 2005) have been used as objects of reading research. While the results thus obtained might be of high ecological validity, they might as well leave ample degrees of freedom for their interpretation (cf. Clifton and Staub, 2011).

What if we seek to combine the benefits of both short textoids and natural reading materials? In case of prose, such a compromise can be found in the construction of vignettes. The term encompasses short, written descriptions of fictitious situations and/or persons (Poulou, 2001). Vignettes usually contain background information and offer readers a base for evaluative judgments (Huebner, 1991; Poulou, 2001). They have been used in the context of teaching (Finch, 1987; Brophy and McCaslin, 1992; Gavrilidou et al., 1993; Poulou, 2001), in the appraisal (Robinson and Clore, 2001), cognitive (Filik and Leuthold, 2013), and emotion recognition research (Camras et al., 1983; Reichenbach and Masters, 1983; Ribordy et al., 1988), to study the theory of mind (Ishii et al., 2004), situational empathy (de Wied et al., 2005), or emotion processing in healthy people (Wilson-Mendenhall et al., 2013), in patients with schizophrenia (Kuperberg et al., 2011), and borderline personality disorder (Levine et al., 1997). Complementary to these applications, the present study evaluates the usefulness of vignettes in the area of emotion induction.

#### Going Beyond Emotion Inferences

In discourse comprehension, vignettes have become prominent stimuli for studying emotional inferences that are specifically concerned with emotions experienced by characters of a story. One often cited set of 24 vignettes was first published by Gernsbacher et al. (1992). The short stories were constructed to examine how readers infer and represent emotional states of a protagonist that are not explicitly mentioned. Since then these stories have been widely used and adapted in further studies investigating the specificity and content of emotional inferences (Gygax et al., 2003, 2004, 2007; Gillioz et al., 2012; Gillioz and Gygax, 2017). It has been shown that emotional inferences are part of readers’ mental representations (Miall, 1989; Graesser et al., 1994), are rather general (e.g., Gygax et al., 2003, 2004), and may only refer to certain parts (e.g., behavioral descriptions) of a multi-componential emotion construct (Gygax et al., 2007; Gillioz and Gygax, 2017).

Most of the above-mentioned studies involved manipulations of a target sentence containing either a matching or mismatching emotion term (e.g., Gygax et al., 2003, 2004) or behavioral description (e.g., Gygax et al., 2007) and focused on the analysis of reading times for target sentences to explore effects of consistent versus inconsistent emotional information. They neither examined emotions elicited in the reader nor compared reactions to positive and negative valences. One exception was published by León et al. (2015). The authors constructed short texts of four sentences possessing either a positive, negative, or neutral valence. The last sentence either ended with a related (e.g., the word *happy* in an emotionally positive context), non-related, or non-word as target word for which a lexical decision had to be performed. Analyses of corresponding reaction times revealed faster reactions to both related and non-related words when presented in an emotionally positive context compared to a negative one.

Notably, effects of emotional valence are likely to go beyond the level of inferences. In a recent study conducted by Megalakaki et al. (2019), native French speakers were instructed to read easily understandable texts varying in their emotional valence and intensity. Subsequently, participants were asked to answer comprehension questions on different levels of discourse (i.e., textbase, surface level, inference level). Analyses of their answers revealed that positive valence facilitated the comprehension of textual contents (surface level) whereas negative valence favored the construction of inferences (inference level). In addition, high emotional intensity promoted the understanding of emotionally positive texts but impeded the comprehension in a negative valence context. Hence, textual valence was found to influence the comprehensibility of reading materials. More importantly, the influential role of valence needs to be considered in eye tracking studies since eye movements have been shown to be affected by both text difficulty (Rayner et al., 2006; Lüdtke et al., 2019) and valence (e.g., Scott et al., 2012; Knickerbocker et al., 2015; Ballenghein et al., 2019).

In the present study, emotionally positive and negative vignettes were constructed to examine their emotion induction potential and analyze effects of emotional content on reading behavior. Although emotional vignettes have been applied in the research field of emotion inferences, valence-specific effects have largely remained unexplored. Moreover, most of the above-mentioned studies made use of the onlooker perspective (i.e., using the pronoun “*he/she”*). However, the personal perspective (i.e., using the pronoun “*you”*) was found to cause both greater internalization of emotional narratives (Brunyé et al., 2011) and stronger effects of positive emotion induction (Child et al., 2020). The thus provoked empathic engagement is assumed to facilitate the presence of immersion (Jacobs, 2015b). While reading, we start forgetting about the physical world around us and feel transported into the book’s fictitious setting. As stated by the Neurocognitive Poetics Model of literary reading (NCPM; Jacobs, 2011, 2015b), immersion leads to faster reading (i.e., shorter fixation, longer saccades) making it directly relevant to the analysis of eye movements. Hence, when examining online reading behavior in emotional vignettes, the immersion potential should be considered as it might interact with both the valence manipulation and the reading behavior.

### Emotion in Picture Viewing

Living in the digital age, we are constantly exposed to pictorial stimuli such as personal photos or social media posts. Related research has highlighted the influential role of both pictures in general and their semantic relevance on attentional processes during visual perception (Pilarczyk and Kuniecki, 2014; Keib et al., 2016). At present, it is widely acknowledged that fixations are biased toward informative regions of our perceptual field (Henderson, 2003). These are areas that either pop out because they are very different with respect to low-level visual features (i.e., high visual saliency) or because they inform about the emotional meaning (i.e., high semantic relevance). When comparing both influential factors, regions of semantic richness tend to attract more fixations than visual salient ones (e.g., Pilarczyk and Kuniecki, 2014). Consequently, eye movements are substantially guided by the distribution of emotional contents (cf. Budimir and Palmović, 2011).

Previous studies examining effects of emotional pictures have stressed processing differences of positive, negative, and neutral pictures (Olofsson et al., 2008, for review). In this context, the privileged processing of emotional stimuli has been explained in terms of their evolutionary and motivational relevance. Although the special role of negatively valenced stimuli (e.g., snakes) has been put forward (e.g., Fox et al., 2000; Yiend and Mathews, 2001; Calvo et al., 2006), the majority of results rather supports the existence of arousal-driven, valence-independent effects. Hence, emotional compared to neutral stimuli were found to initially attract and maintain attentional processes (Calvo and Lang, 2004; Calvo and Avero, 2005; Nummenmaa et al., 2006; Carniglia et al., 2012).

With respect to the present study, results from previous eye tracking experiments using a free viewing paradigm with pictures presented one at a time are of particular interest as this is the paradigm used for emotion induction. Eye movements can serve as an indicator for (overt) attentional processes since both are tightly coupled. Thus, an attentional shift is usually linked to the execution of saccades (Findlay and Gilchrist, 2003). To the best of our knowledge, only a handful of eye tracking studies made use of the above-mentioned paradigm applied in the area of emotion induction (Christianson et al., 1991; Bradley et al., 2008, 2011; Niu et al., 2012; Yang et al., 2012; Lanatà et al., 2013; Henderson et al., 2014). Among them, only two allow for the comparison of eye movements on positively and negatively valenced pictures. In this context, Bradley et al. (2011) presented emotionally charged and neutral pictures for a free viewing period of 6 seconds (s). Eye movements were analyzed in terms of three parameters: number of fixations, average fixation duration, and total scan path (i.e., length of all saccades). Their results showed that emotional compared to neutral pictures possessed longer scan paths and attracted more as well as shorter fixations. No valence-specific effects on eye movements were found. Niu et al. (2012) addressed the research question to which extent gaze behaviors are driven by visually salient versus affective features. Consequently, their analyses were performed on the level of pre-defined areas of interest. High arousal proved to increase the probability of fixations on affective regions independent of the pictorial valence.

In sum, while indicating differences between affective and neutral pictures, studies with a comparable experimental design suggest an absence of valence-specific effects on eye movements in healthy participants. It should be noted that the majority of the above-mentioned studies worked with pictures from the *International Affective Picture System* (IAPS; Lang et al., 2008). Despite the widespread use of the cross-validated database, three associated issues should be considered (Dan-Glauser and Scherer, 2011; Marchewka et al., 2014). First, the vast majority of pictures contains people as primary objects limiting its usefulness when studying the influence of content-specific effects. Second, due to its frequent application, processes of familiarity might occur and possibly reduce emotion-inducing effects. Third, some stimuli are outdated and of lower visual quality. To address these issues, a comprehensive, modern alternative is provided by the NAPS (Marchewka et al., 2014).

### Emotion Induction: The Role of Stimulus Domains

So far, evidence supporting the privileged sensory and cognitive processing of emotional compared to neutral stimuli has been provided for both pictorial and verbal materials. However, pictures have been claimed to induce stronger emotional reactions than words (e.g., De Houwer and Hermans, 1994; Larsen et al., 2003; Bayer and Schacht, 2014, for review). This view has largely been supported by evidence from EEG (e.g., Azizian et al., 2006) and fMRI (e.g., Kensinger and Schacter, 2006) studies stressing temporal and topographical differences. According to dual coding theories (e.g., Glaser, 1992), pictorial and verbal materials vary with respect to their processing channels and semantic representations. In this context, the reported superior processing of pictures compared to words has been explained by their more direct access to the semantic system (e.g., Seifert, 1997). Hence, linguistic stimuli, as abstract and learned symbols, were assumed to require additional translational processes for the extraction of meaning (cf. Schlochtermeier et al., 2013). With respect to emotional valence, different processing biases have been reported for pictures and words (Bayer and Schacht, 2014). More precisely, pictures were associated with a negativity bias whereas verbal stimuli were claimed to show a preferential processing of positive valence.

Notably, differences between both stimulus domains have mostly been reported when the processing of mere perceptual features was sufficient to perform the task (e.g., Pegna et al., 2004; Hinojosa et al., 2009; Schacht and Sommer, 2009a; Rellecke et al., 2011). In this connection, many studies neglected to collect evaluative judgments and thus missed the analysis of perceived emotion effects. In contrast, when semantic processing was demanded, a comparable effectivity of both stimulus domains has been put forward (Schacht and Sommer, 2009a; Schlochtermeier et al., 2013; Tempel et al., 2013; Bayer and Schacht, 2014). For example, the EEG study by Bayer and Schacht (2014) compared effects of emotional words and pictures (positive, negative, neutral) using a recognition memory task. Both stimulus domains elicited emotion effects at early and late processing stages. Besides, collected ratings of valence revealed that words were perceived as more pleasant within the groups of positive and neutral stimuli. For arousal, no main effect of stimulus domain was found. Hence, words were not rated as less arousing in general. Interestingly, when reducing the complexity of pictures (e.g., by using black-white pictograms), superior emotion effects of words compared to their pictorial counterparts were reported (Schlochtermeier et al., 2013; Tempel et al., 2013). In these imaging studies (i.e., EEG, fMRI), effects of emotionally positive and neutral stimuli were examined for both materials (i.e., pictures, words) while accounting for differences in stimulus complexity. At the neural level, words were found to elicit more widespread and larger activities than pictures. Moreover, positively valenced words attracted faster (Tempel et al., 2013) as well as stronger (Schlochtermeier et al., 2013) subjective ratings of emotional valence.

Most of the above-mentioned studies applied words, especially nouns (e.g., Hinojosa et al., 2009, 2010; Bayer and Schacht, 2014), to represent the verbal stimulus domain. However, processing differences between words and pictures might be partly mediated by effects of stimulus complexity. When controlling for this confounding factor by using more complex linguistic materials (e.g., phrases), processing differences incline to disappear (Schlochtermeier et al., 2013). In sum, previous studies suggest that both the task demand and the stimulus complexity are of crucial role when comparing the emotion induction potential of verbal stimuli and pictures (e.g., Hinojosa et al., 2010; Bayer and Schacht, 2014). To the best of our knowledge, this is the first article including the direct (i.e., within-subject design) comparison of emotional vignettes and pictures with shared semantic content. Moreover, the present study addresses the suggested importance for individual ratings being recorded within the same group of participants as further variables of interest (e.g., physiological activity). For example, it has been shown that evaluative judgments of arousal differ from provided normative ratings (Olofsson et al., 2008). In line with this perspective, we aimed to operationalize valence-specific effects through the perceived (i.e., subjective ratings) and not experimentally manipulated (i.e., positive, negative) valence. Hence, individual differences in the perception of emotional stimuli were anticipated and accounted for.

### Hypotheses

The present study aimed to examine effects of emotional materials (i.e., vignettes, pictures) on (1) ratings of Perceived Valence and Arousal, (2) eye movements in reading, and (3) eye movements in picture viewing. We therefore selected 40 emotionally valenced (i.e., positive, negative) pictures and vignettes, respectively. We assumed that our valence manipulation would influence subjective ratings of both Perceived Valence and Arousal. More specifically, based on the strongly negative, linear relationship between valence and arousal reported for the consulted NAPS database (Marchewka et al., 2014), we expected that emotionally positive stimuli (i.e., vignettes, pictures) would, on average, be rated more positively (i.e., Perceived Valence) and as less arousing (i.e., Perceived Arousal) than emotionally negative ones.

Based on prior findings indicating stronger valence effects of emotionally positive words compared to pictures (e.g., Schlochtermeier et al., 2013; Tempel et al., 2013; Bayer and Schacht, 2014), we also suggested that emotionally positive vignettes would, on average, be rated more positively than emotionally positive pictures. Moreover, we assumed that this domain-specific effect would also apply to the negative valence category. Hence, we expected that emotionally negative vignettes would, on average, be perceived more negatively than emotionally negative pictures. With respect to subjective ratings of Perceived Arousal, there is evidence that words are able to induce arousal levels that are comparable to the ones elicited through pictures (e.g., Schlochtermeier et al., 2013; Bayer and Schacht, 2014). Consequently, it was assumed that stimulus domains (i.e., textual, pictorial) would not differ in their induced arousal levels.

With respect to effects of Perceived Valence on eye movements in reading of ecologically valid stimuli, reading times for both vignettes and their constituting words were of primary interest. Based on previous results showing faster processing of emotionally positive words, sentences, and texts (e.g., Kousta et al., 2009; Briesemeister et al., 2011; Lüdtke and Jacobs, 2015; Ballenghein et al., 2019), we assumed that vignettes perceived as emotionally positive would, on average, attract shorter reading times than vignettes perceived as emotionally negative. In this context, the first eye tracking study examining valence-specific effects at the supralexical level indicated shortest reading times for positive, followed by negative, and lastly neutral narratives (Ballenghein et al., 2019).

In line with reported correlations between lexical and textual valence ratings (Bestgen, 1994; Whissell, 2003; Hsu et al., 2015b; Jacobs, 2015b), we expected that Perceived Valence would likewise affect reading times at the lexical level (i.e., words). Since our study refers to effects of an affective semantic superfeature (valence; Jacobs et al., 2016a), content words were of main interest (cf. Bestgen, 1994). Thus, we expected that content words constituting vignettes perceived as emotionally positive would, on average, attract shorter reading times than content words constituting vignettes perceived as emotionally negative. Since it remained inconclusive at which processing stage valence-specific effects on reading times for lexical units (i.e., words) would become evident, eye tracking measures reflecting both early (e.g., first fixation duration) and later (e.g., word total reading time) processes were considered (cf. Lüdtke et al., 2019).

Lastly, we aimed to examine the influence of Perceived Valence on eye movements during picture viewing. In line with previous eye tracking studies suggesting an absence of valence-specific effects (e.g., Bradley et al., 2011; Niu et al., 2012), we assumed that pictures perceived as emotionally positive would attract scan (e.g., Mean Saccade Amplitude) and fixation (e.g., Total Number of Fixations) patterns that are similar to the ones provided by pictures perceived as emotionally negative.

## Materials and Methods

In order to examine the above-stated hypotheses, eye movements of 42 participants were recorded while reading and viewing 40 emotional vignettes and pictures, respectively. Textual stimuli were constructed based on pre-selected emotional pictures and validated in two pilot studies. For both material groups, stimuli were presented one at a time and followed by an evaluative judgment task which required participants to assess the emotional valence and arousal of each stimulus. Accordingly, linear mixed-effects models were performed to analyze effects of valence (i.e., Valence Category, Valence Rating) and Stimulus Domain on (1) ratings of Perceived Valence and Arousal, (2) eye movements in reading, and (3) eye movements in picture viewing. Since the present study was designed to address effects of emotion induction, individual ratings of valence (and arousal) were used to define the emotional quality of our stimuli (cf. Rubo and Gamer, 2018).

### Participants

Forty-two native German speakers (33 female, 1 non-binary; *M*_age_ = 23.81 years, *SD*_age_ = 5.41, age range: 18–44 years) gave their informed, written consent for participation and further use of their anonymized data. They were recruited through collegiate tutorials in the Bachelor’s degree of Psychology at Freie Universität Berlin as well as from announcements on social media. Participants either received course credit (88.1%) or took part voluntarily. All of them had normal or corrected-to-normal vision. Thirty-three participants (78.6%) named a general qualification for university entrance as highest level of education. The study was approved by the ethics committee of the Department of Education and Psychology at Freie Universität Berlin.

### Recording of Eye Movements

Eye movements were recorded with an SR Research EyeLink 1000 tower-mounted eye tracker providing a sampling rate of 1000 Hz (SR Research Ltd., Mississauga, ON, Canada). Due to the chin-and-head rest, head movements could be reduced to a minimum. Recording of eye movements occurred exclusively during stimulus presentation in which only the right eye was tracked. The experiment was built using the SR Research Experiment Builder software (version 1.10.1630)^1^. Stimuli were presented on a 19-inch LCD monitor with a resolution of 1024 × 768 pixels and a refresh rate of 120 Hz. The distance between the participant’s eyes and the monitor measured approximately 65 centimeters. At the beginning of the experiment, a standard 9-point calibration was used to ensure a spatial resolution error of less than 0.5° of visual angle. To avoid a permanent repetition of this time-consuming and distracting procedure, each reading and viewing trial started with two sequentially presented fixation crosses (Times New Roman, 20 point-size). They were either positioned above the first reading line at the right and left corner of the display or arranged at the upper right and left corner of the subsequently presented picture. For each of them, a rectangular area of interest (AOI; 70 × 62 pixels) was defined. When a total fixation time of 500 milliseconds (ms) was registered in each AOI, stimulus presentation started automatically. Fixations and saccades were identified using the EyeLink 1000 parser (velocity threshold = 30°/sec, acceleration threshold = 8000°/sec^2^).

### Stimuli

Emotional stimuli were selected and constructed following a stepwise procedure. As previously stated, pictures and vignettes were intended to provide comparable semantic information. Hence, the construction process started with the collection of 48 emotion-inducing pictures (24 emotionally positive, 24 emotionally negative) from the NAPS (Marchewka et al., 2014). The standardized, high-quality database includes normative ratings for over 1,000 realistic pictures. A major advantage for eye tracking studies concerns the availability of information on physical properties. Since eye movements are known to be affected by low-level visual features such as complexity, luminance, or contrast (e.g., Bradley et al., 2007; Pilarczyk and Kuniecki, 2014), we aimed to control for these confounding factors. In sum, the following inclusion criteria were applied: First, pictures had to possess normative valence ratings either below four or above six^2^ to minimize the potential overlap between both valence categories. Second, valence categories were not allowed to vary with respect to the following physical parameters: luminance, contrast, JPEG size, color composition (i.e., LABL, LABA, LABB), entropy, and format (landscape; 1600 × 1200 pixels). Third, valence categories had to consist of pictures similarly distributed among the provided content categories [i.e., animals, faces, people, objects, landscapes; for the final stimulus set: χ^2^(3,*N* = 40) < 1, *p* = 0.97, *R*^2^ < 0.01].

Based on the selected pictures, 48 vignettes verbally reproducing the pictorial information were constructed by the first author and Ilai Jess. To avoid a systematic influence of the narrative perspective, readers were continuously addressed in the second person singular (e.g., Miall and Kuiken, 2001; Brunyé et al., 2009). The text length was kept between 85 and 96 words. To ensure a high comprehensibility and emotion induction potential, an online pilot study was conducted via SoSci Survey (Leiner, 2019)^3^. Fifty-three people were recruited from announcements on social media and either received course credit or participated voluntarily (33 female, 15 non-binaries; *M*_age_ = 33.81 years, *SD*_age_ = 14.56, age range: 17–71 years). Questions referring to Valence, Arousal, Comprehensibility, Immersion Potential, and Emotion Induction Potential were rated after self-paced reading of the randomly ordered 48 vignettes (24 emotionally positive, 24 emotionally negative). Valence and Arousal were rated on a 9-point rating scale, for the three other measures 5-point rating scales were applied.

In a next step, potentially problematic vignettes were identified based on the average valence ratings. In consideration of Comprehensibility and the physical parameters of the corresponding pictures, eight vignettes were excluded, nine additional ones revised and rated for a second time (*N* = 13, *M*_age_ = 35.69 years, *SD*_age_ = 11.39, age range: 22–60 years). Table 1 includes the descriptive statistics of the final stimulus set (20 emotionally positive, 20 emotionally negative vignettes), Table 2 entails an example for each valence category. Information on the corresponding pictures can be found in Table 3. The results indicated that the vignettes are easy to understand and capable of inducing negative and positive emotional responses. Furthermore, positive and negative valence groups showed differences on the rated dimensions. Emotionally negative vignettes were, on average, rated higher with respect to Arousal [*t*(38) = −11.95, *p* < 0.001, *R*^2^ = 0.79] and Emotion Induction Potential [*t*(38) = −4.04, *p* < 0.001, *R*^2^ = 0.30] whereas positive ones seemed to be easier to understand [*t*(38) = 2.52, *p* = 0.02, *R*^2^ = 0.14] and better suited to put the reader in the perspective of the text [*t*(38) = 2.73, *p* < 0.01, *R*^2^ = 0.16]. Most importantly, valence ratings supported the success of our valence manipulation: emotionally positive vignettes were, on average, perceived more positively than emotionally negative ones [*t*(38) = 33.97, *p* < 0.001, *R*^2^ = 0.97]. As expected, ratings of Valence and Arousal possessed a strong negative linear correlation [*r* = −0.91, *t*(38) = −13.36, *p* < 0.001, *R*^2^ = 0.82]. This negative correlation could also be observed between Valence and Arousal ratings reported for the NAPS pictures [*r* = −0.96, *t*(38) = −20.9, *p* < 0.001, *R*^2^ = 0.92].

**TABLE 1:** Statistical parameters for the emotional vignettes.

	***Valence Category***					
	**Positive**		**Negative**			
**Rating Dimension**	***M***	***SD***	***M***	***SD***	***t-*value^1^**	***R*^2^**
Valence^2^	7.15	0.38	2.53	0.48	33.97***	0.97
Arousal^2^	4.20	0.71	6.40	0.41	−11.95***	0.79
Comprehensibility^3^	4.82	0.08	4.75	0.09	2.52*	0.14
Immersion Potential^3^	4.29	0.15	4.16	0.15	2.73**	0.16
Emotion Induction Potential^3^	3.82	0.25	4.14	0.26	−4.04***	0.30

^1^Pooled *t*-tests (*df* = 38) were run in JMP Pro 14. Analyses were performed on aggregated mean values. ^2^Valence and Arousal were rated on a 9-point rating scale. ^3^Comprehensibility, Immersion Potential, and Emotion Induction Potential were rated on a 5-point rating scale. *p*-values were denoted as follows: *p < 0.05, **p < 0.01, ***p < 0.001.

**TABLE 2:** Examples of the self-constructed, emotion-inducing vignettes.

**Valence Category**	**German**	**English**	**Picture ID^1^**
Negative	Das kleine Rehkitz liegt wie versteinert in der blutverschmierten Plastikplane vor dir. Seine Augen sind vor Angst und Panik weit aufgerissen und blicken unbeweglich auf den Boden. Ein Wolf hat seine Mutter angefallen und es dabei verletzt. Sein Hinterbein ist aufgerissen und der Angreifer hat eine große klaffende Wunde hinterlassen. Es sieht aus, als wäre dem kleinen Rehkitz ein Stück Fell abgezogen worden. Der Wolf hat ihm ein großes Stück Fleisch aus dem Hinterbein gerissen, ehe er es liegen gelassen hat, um die Mutter des Rehkitzes zu töten.	The little fawn lies petrified in front of you in the blood-stained plastic tarpaulin. Its eyes are wide open from fear and panic and look motionless at the ground. A wolf has attacked his mother and injured her. Its hind leg is torn open and the attacker has left a large gaping wound. It looks as if a piece of fur has been peeled off the little fawn. The wolf tore a large piece of flesh from his hind leg before he left it to kill the fawn’s mother.	Animals_063_h
Positive	Es ist ein entspannter Sonntagmorgen. Du konntest ausschlafen und hast dir nun in aller Ruhe ein genussvolles Frühstück zubereitet. Du stellst alles auf deinen Holztisch in der Küche und setzt dich munter auf einen der Stühle. Du freust dich auf deinen morgendlichen Kaffee und betrachtest dein gesundes Frühstück: zwei Mehrkornbrotscheiben mit Käse, roten Tomaten und frischem Basilikum. Erst gestern hast du das Brot selbst gebacken. Der Kaffee ist noch heiß und du gießt Milch dazu. Nun kannst du unbeschwert dein Essen genießen und anschließend gestärkt in den Tag starten!	It is a relaxed Sunday morning. You were able to sleep well and have now prepared a delicious breakfast in peace and quiet. You put everything on your wooden table in the kitchen and sit down on one of the chairs. You look forward to your morning coffee and consider your healthy breakfast: two slices of multigrain bread with cheese, red tomatoes, and fresh basil. Only yesterday you baked the bread yourself. The coffee is still hot, and you pour milk on it. Now you can enjoy your meal and start the day strengthened!	Objects_097_h

*Vignettes were constructed using 40 pictures from the *Nencki Affective Picture System* (NAPS; Marchewka et al., 2014). ^1^Picture ID refers to the corresponding ID from the NAPS.*

**TABLE 3:** Statistical parameters for the emotional pictures.

	**Valence Category**					
	**Positive**		**Negative**			
**Dimension**	***M***	***SD***	***M***	***SD***	***t*-value^1^**	***R*^2^**
*Ratings*^2^						
Valence	7.52	0.18	2.52	0.35	56.33***	0.99
Arousal	4.42	0.45	6.87	0.37	−18.86***	0.90
*Physical parameters*^3^						
Contrast	61.09	10.96	66.98	7.73	–1.96	0.09
Entropy	7.54	0.25	7.66	0.25	–1.47	0.05
JPEG size	310837.30	123130.90	312743.35	95487.89	–0.05	<0.01
Luminance	119.31	33.27	104.84	20.42	1.66	0.07
LABL	49.45	13.04	43.56	8.52	1.69	0.07
LABA	3.03	6.97	4.15	5.75	–0.55	0.01
LABB	7.75	6.34	7.86	7.49	–0.05	<0.01

^1^Pooled *t*-tests (*df* = 38) were run in JMP Pro 14. Analyses were performed on aggregated mean values. ^2^Valence and Arousal were rated on a continuous sliding scale (range: 1–9; Marchewka et al., 2014). Values are based on the entire norm sample. ^3^Further information is provided by Marchewka et al. (2014). *p*-values (if *p* < 0.05) were denoted as follows: ***p < 0.001.

To establish comparable initial situations in the eye tracking experiment, three emotionally neutral vignettes^4^ were additionally constructed based on corresponding neutral NAPS pictures (characterized on average by the same physical parameters as the emotional pictures; Valence: *M* = 4.52, *SD* = 0.4; Arousal: *M* = 5.36, *SD* = 0.45). The entire set of constructed vignettes including the names of their corresponding NAPS pictures is provided in the Supplementary Tables S1–S3.

### Design and Procedure

A repeated measures design was implemented (i.e., each subject viewed and read the entire stimulus set). Stimulus domains were presented in separate blocks. The order of blocks was counterbalanced across participants. Within each block, stimuli were presented in a pseudo-randomized sequence so that no more than two stimuli of the same valence category were presented successively.

The study was conducted in a sound-attenuated room separated from the daylight. After arriving, participants were informed about the procedure as well as the option to quit the experiment at any time without facing any consequences. The experiment started with a standard 9-point calibration and was followed by a sequential presentation of three emotionally neutral stimuli in order to match the initial situation between participants. Afterward, the participant’s mood was measured on a 7-point, non-verbal rating scale offering the possibility to account for mood differences^5^. Ratings indicated a slightly positive mood before the presentation of both emotional vignettes (*M* = 5.26, *SD* = 0.59) and pictures (*M* = 5.19, *SD* = 0.71).

Subsequent to the rating, emotional stimuli of the first block (i.e., vignettes or pictures) were presented. Each trial started with two sequentially appearing fixation crosses which had to be looked at for 500 ms, respectively. For the vignettes, reading speed was self-controlled allowing participants to go back and forth within a single page as often as they wanted. Each vignette was presented on a single page (eight to eleven lines) and could be left by a single mouse click. Vignettes were written in a sans-serif font (Tahoma) with 17-point letter size and presented left-aligned in the center of the monitor. In order to maximize the accuracy of the recordings, double-spacing was used. Participants were instructed to read each story for comprehension. Pictures (800 × 600 pixels) were presented for a fixed viewing period of 3 s in the center of the display. Participants were instructed to freely look at each picture for the whole presentation time. After each stimulus presentation, participants were instructed to perform an evaluative judgment task. More specifically, subjects were asked to assess each stimulus in terms of its emotional valence (i.e., *How positive or negative do you rate the text/picture?*) and arousal (i.e., *How calming or exciting do you rate the text/picture?*). Answers were given on 9-point Self-Assessment-Manikin scales (SAM; Lang, 1980; Suk, 2006). Rating scales were displayed sequentially in the center of the monitor. No time restrictions were provided.

**FIGURE 1:**
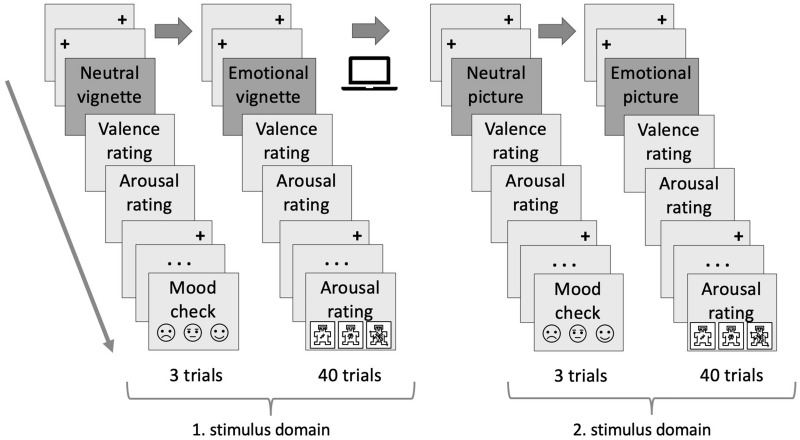
Graphic depiction of the experimental procedure. Participants performed an evaluative judgment task on textual and pictorial stimuli, respectively. The order of stimulus domains (i.e., pictorial, textual) was balanced across participants. To ensure comparable initial situations, each experimental session started with the presentation of three emotionally neutral stimuli. At the beginning of each trial, two serially presented fixation crosses had to be looked at for 500 ms. After assessing participants’ mood, 40 emotionally valenced stimuli were sequentially displayed while recording eye movements. Pictures were viewed for a fixed period of 3 s. Reading of vignettes was self-controlled. Ratings of perceived valence and arousal were measured using a 9-point Self-Assessment-Manikin scale (SAM; Lang, 1980; Suk, 2006). After completion of the first presentation block, an online survey was conducted.

When finished with the first block, an online survey referring to demographic variables, reading habits, imagination, and empathy was answered at a separate table. At their own free discretion, participants returned to the eye tracker and completed the same procedure on the remaining stimulus domain starting again with the 9-point calibration. In sum, an experimental session lasted approximately 60 minutes. Figure 1 provides an illustration of the experimental procedure.

### Data Analysis

In line with the tripartite structure of our hypotheses, the following sections will be arranged into three subparts: (1) analysis of evaluative judgments, (2) analysis of eye movements in reading, and (3) analysis of eye movements in picture viewing. All analyses refer to data for the emotionally positive and negative stimuli. Due to their experimental function and the limited number, data for the emotionally neutral vignettes and pictures will only be reported in terms of descriptive statistics. However, the same preprocessing steps were applied.

### Data Preprocessing

For the 40 emotional vignettes and pictures, we collected 3,360 ratings for both Perceived Valence and Arousal (42 participants à 80 ratings). As two trials had to be excluded due to errors during the export of data, 3,358 individual ratings and reaction times for each subject and item (i.e., pictures, vignettes) could be used for statistical analysis.

Eye tracking data were preprocessed using the EyeLink Data Viewer (version 1.11.900)^6^. Fixations less than 80 ms were either merged with nearby fixations (distance of less than one degree) or removed from further analysis. Based on automatically defined AOIs, text data were exported on the level of single words (150,899 data points). Further aggregation of data and preprocessing were run in JMP Pro 14 for Mac OS X^7^. The selection of eye tracking parameters resulted from our hypotheses on reading times for both supralexical (i.e., vignettes) and lexical units (i.e., words). For the analysis at the supralexical level, text total reading time as the sum of all fixations, saccadic movements, and blinks was computed. For the analysis at the lexical level, we aimed to study a measure associated with early and a measure associated with both early and late processes of word recognition and comprehension (cf. Lüdtke et al., 2019). To analyze immediate effects of Perceived Valence, the commonly reported duration of the first fixation on each word was extracted (Hyönä et al., 2003; Kuperman and Van Dyke, 2011). As late measure, word total reading time (afterward called total reading time, TRT) defined as the total sum of all fixation durations on a word was used (Boston et al., 2008). Since valence groups of emotionally positive and negative vignettes varied slightly in their text lengths [*t*(38) = 2.07, *p* = 0.046, *R*^2^ = 0.10], we accounted for the difference by computing Reading Speed [in words per minute (wpm)], mean First Fixation Duration (mean FFD in ms), and mean Total Reading Time (mean TRT in ms) for each subject and vignette.

To calculate mean FFD and mean TRT, we first excluded all function words (articles, pronouns, conjunctions, auxiliary verbs, prepositions, particles, cardinal numbers, pronominal adverbs) as they lack or are poor in lexical or affective lexical meaning (cf. Segalowitz and Lane, 2000; Fiedler, 2011). Part-of-speech (POS) tagging was automatized using the freeware tagger TagAnt (Anthony, 2015). Like any POS tagger TagAnt produces error rates of approximately three percent (Manning, 2011), and thus obviously wrong classifications were corrected by hand. On the remaining 75,348 data points (see Table 4), extreme values were defined and excluded following a two-stage procedure. First, FFDs larger than 2,000 ms (six data points) and TRTs larger than 3,000 ms (five data points) were excluded. Second, outliers were defined based on the distributions of FFDs and TRTs within each subject and vignette. Words with standardized residuals larger or smaller than three were excluded [FFD: 655 data points (0.87%); TRT: 1,174 data points (1.56%)]. Based on the remaining data points, mean FFD and mean TRT were computed for each subject and vignette treating skipped words (mean skipping: 19.65%) as missing values. Taken together, the resulting data table contained 1,678 data points including information about mean FFD (in ms), mean TRT (in ms), and Reading Speed (in wpm) for each subject and item.

**TABLE 4:** Number and percentage of content, function, and skipped words within each valence category.

	**Valence Category**		
	**Positive**	**Negative**	**Neutral**
*Function words*			
Number of data points	37,548	38,003	5,376
Percentage^1^	49.12	51.04	47.41
Number of unique words	112	128	64
*Content words*			
Number of data points	38,892	36,456	5,964
Percentage^1^	50.88	48.96	52.59
Number of unique words	570	585	128
*Skipped words*			
Number of data points	26,939	25,472	3,158
Percentage^1^	35.24	34.21	27.85

*The data table consisted of 150,899 data points (i.e., each row represented one word). ^1^Percentage relative to the total number of data points belonging to the positive, negative, or neutral valence category, respectively.*

With respect to the pictures, eye tracking data were exported on the level of trials for each participant (42 participants à 40 trials; 1,680 data points). Since we aimed to investigate whether effects of Perceived Valence are reflected in measures of both fixations and saccades, the selection of eye tracking parameters was inspired by Bradley et al. (2011). Hence, statistical analyses were performed on Mean Saccade Amplitude (i.e., the distance between two consecutive fixations; Radach and Kennedy, 2013), Total Number of Fixations (i.e., number of fixations within the viewing period of 3 s), and Mean Fixation Duration.

### Statistical Analysis

All statistical analyses were run in R 3.5.1 for Mac OS X^8^. Since participants and items (i.e., vignettes, pictures) represented samples of larger populations, hypotheses were tested using linear mixed-effects models (LMM; Baayen et al., 2008). Following a confirmatory approach on real data, intercepts-only models with by-item and by-subject random intercepts were employed (cf. Bates et al., 2015a). In R, models were computed using the lmer-function from the lme4 package (Bates et al., 2015b) with restricted maximum likelihood estimation.

To obtain the optimal fixed-effects structure (i.e., trade-off between fit to the data and complexity), models were selected according to a backward-elimination procedure (cf. Barr et al., 2013). Starting with random-intercepts models accounting for all possible fixed-effects terms, predictor variables were successively excluded based on the strongest evidence (i.e., highest *p*-value). If the variable with the strongest evidence was involved in an interaction with less evidence, the predictor with the second highest evidence was excluded. In an iterative procedure, nested models differing in one degree of freedom (i.e., one fixed effect) were systematically compared using the anova-function from the stats package (R Core Team, 2019). To justify a reduction of fixed-effects terms, likelihood ratio tests were performed. Decisions were based on the statistical significance (*p <* 0.05) of the asymptotically chi-squared distributed likelihood ratio test statistic with one degree of freedom. If the likelihood of the simpler model was not significantly worse than the likelihood of the more complex model (*p >* 0.05), the former was favored.

For the analysis of evaluative judgments (i.e., Perceived Valence and Arousal), the following predictor variables were initially included: Valence Category (i.e., positive, negative), Stimulus Domain (i.e., pictorial, textual), and Mood Rating. To test for valence-specific effects, the interaction between Valence Category and Stimulus Domain was included. For the analysis of reading behavior at the supralexical (i.e., Reading Speed) and lexical level (i.e., mean FFD, and mean TRT), initial models consisted of the following predictor variables: Perceived Valence (i.e., Valence Rating), Perceived Arousal (i.e., Arousal Rating), Mood Rating, and three characteristics of the vignettes collected in the pilot studies (Comprehensibility, Immersion Potential, Emotion Induction Potential). Considering theoretically and empirically provided evidence (e.g., Võ et al., 2009; Marchewka et al., 2014), we included the interaction between Perceived Valence and Arousal. For the analysis of picture viewing (i.e., Mean Saccade Amplitude, Total Number of Fixations, and Mean Fixation Duration), the following predictor variables were initially included: Perceived Valence (i.e., Valence Rating), Perceived Arousal (i.e., Arousal Rating), and Mood Rating. Again, the interaction between Perceived Valence and Arousal was included. Detailed information on the mathematical formulation and lmer specification of all eight initial models are reported in the Supplementary Tables S4–S11.

For the categorical variables (i.e., Valence Category, Stimulus Domain), effect coding was chosen. The metrical covariates were centered prior to analysis in order to avoid collinearity, increase probability of model convergence, and facilitate interpretations (Baayen, 2008). Fixed effects were checked with Type III sum of squares statistics using the Anova-function from the car package (Fox and Weisberg, 2019). To ensure a best possible approximation of the residuals’ distribution to the normal distribution, dependent variables were transformed as indicated by the Box-Cox transformation test from the MASS package (Box and Cox, 1964; Venables and Ripley, 2002). For all eye tracking variables, exclusion of extreme values followed a stepwise procedure. First, an absolute criterion in form of an upper threshold was applied based on the visual inspection of the distribution of each dependent variable. Second, extreme values were defined based on intercepts-only models including only crossed random effects for subjects and items. Since no missing values existed, the relative criterion was set to two standard deviations from the mean. For the evaluative judgments, extreme values were defined based on the recorded reaction times (RTs in ms). The lower and upper thresholds were set to 500 and 20,000 ms, respectively. All statistical analyses are based on a 95% level of significance (α = 0.05). For the sake of conciseness, only fixed effects of the best-fitting model will be reported as they are directly relevant to our hypotheses. Results of the entire stepwise deletion procedure are provided in the Supplementary Tables S4–S11.

## Results

### Descriptive Statistics

To illustrate effects of the experimental manipulation, descriptive statistics are provided for each valence category (i.e., positive, negative, neutral) and stimulus domain (vignettes: Table 5, pictures: Table 6). As expected, ratings of Perceived Valence and Arousal differed between valence categories. For both stimulus domains, lowest mean valence ratings were observed for the negative, followed by the neutral, and lastly the positive valence category. With respect to mean arousal ratings, the following rank order became evident for vignettes and pictures: positive < neutral < negative. As indicated by the minimum and maximum values, each valence category attracted a wide range of individual ratings on both scales. For example, negatively valenced pictures attracted subjective valence ratings ranging from one to eight, with the maximum value indicating a perceived positive valence (see Table 6). For both stimulus domains, correlations between ratings of Perceived Valence and Arousal were more pronounced within the negative valence category [pictures: *r*_negative_ = −0.61, *t*(838) = −22.34, *p* < 0.001, *R*^2^ = 0.37; vignettes: *r*_negative_ = −0.50, *t*(836) = −16.74, *p* < 0.001, *R*^2^ = 0.25] than within the positive one [pictures: *r*_positive_ = 0.09, *t*(838) = 2.57, *p* = 0.01, *R*^2^ < 0.01; vignettes: *r*_positive_ = 0.05, *t*(838) = 1.53, *p* = 0.13, *R*^2^ < 0.01]. The overall correlations indicated a highly negative linear correlation between Perceived Valence and Arousal for both pictures and vignettes [pictures: *r*_overall_ = −0.66, *t*(1678) = −35.78, *p* < 0.001, *R*^2^ = 0.43; vignettes: *r*_overall_ = −0.67, *t*(1676) = −36.80, *p* < 0.001, *R*^2^ = 0.45].

**TABLE 5:** Descriptive statistics for the vignettes based on the eye tracking study.

	**Valence Category**								
	**Negative (*N* = 20)**			**Positive (*N* = 20)**			**Neutral (*N* = 3)**		
**Dependent variable**	***M***	***SD***	**Min-Max**	***M***	***SD***	**Min-Max**	***M***	***SD***	**Min-Max**
*Supralexical level*									
Valence rating^1^	2.05	0.97	1–7	7.61	1.07	3–9	5.49	1.15	3–8
Arousal rating^1^	6.45	1.81	1–9	3.09	2.03	1–9	3.32	1.90	1–7
Reading speed	361.27	129.95	132.89–1262.69	373.68	117.45	159.76–862.33	293.71	84.96	166.34–585.86
*Lexical level*^2^									
Mean FFD	197.05	24.59	141.32–287.11	196.88	25.36	145.27–313.11	207.64	21.17	159.63–266.46
Mean TRT	272.36	59.97	152.24–565.34	262.14	56.86	145.18–516.93	297.71	51.04	192.88–423.57

*Descriptive statistics were computed based on aggregated values for each participant and vignette. ^1^Ratings were assessed on 9-point rating scales. ^2^Analyses for mean First Fixation Duration (mean FFD in ms) and mean Total Reading Time (mean TRT in ms) included only content words.*

**TABLE 6:** Descriptive statistics for the pictures based on the eye tracking study.

	**Valence Category**								
	**Negative (*N* = 20)**			**Positive (*N* = 20)**			**Neutral (*N* = 3)**		
**Dependent variable**	***M***	***SD***	**Min-Max**	***M***	***SD***	**Min-Max**	***M***	***SD***	**Min-Max**
*Ratings*									
Valence^1^	2.2.	1.11	1–8	7.33	1.28	1–9	4.80	1.44	2–9
Arousal^1^	6.42	1.93	1–9	3.11	2.00	1–9	3.65	1.88	1–7
*Eye tracking*									
Total number of fixations	10.89	1.86	4–17	10.37	2.08	3–15	10.16	2.30	3–14
Mean fixation duration^2^	297.29	77.70	176.14–928.75	323.15	112.20	163.21–1226	350.56	167.85	200.38–1279.67
Mean Saccade amplitude^3^	5.64	1.31	2.61–10.49	4.99	1.26	2.28–12.56	5.33	1.43	2.74–9.23

*Descriptive statistics were computed based on aggregated values for each participant and picture. ^1^Ratings were assessed on 9-point rating scales. ^2^Mean Fixation Duration was measures in ms. ^3^Mean Saccade Amplitude was measured in degrees (°) of visual angle.*

With respect to the supralexical eye tracking parameter Reading Speed, we observed fastest reading for positive, followed by negative, and lastly emotionally neutral vignettes. The same rank order was observed at the lexical level (i.e., mean FFD, mean TRT). Regarding the pictorial stimuli, average values of Mean Saccade Amplitude were shortest for the positive, followed by the neutral, and lastly negative valence category. For Mean Fixation Duration, average values suggested the following rank order: negative < positive < neutral. All valence groups attracted, on average, ten to eleven fixations.

### Evaluative Judgments

Mean RTs for Valence Rating did not significantly differ between pictorial (*M* = 2417.23, *SD* = 1436.76) and textual (*M* = 2491.15, *SD* = 1841.54) materials [*t*(3356) = 1.30, *p* = 0.19, *R*^2^ < 0.01]. For Arousal Rating [*t*(3356) = 2.03, *p* = 0.04, *R*^2^ < 0.01], pictures (*M* = 2413.37, *SD* = 2032.61) were, on average, rated faster than vignettes (*M* = 2547.64, *SD* = 1800.41). Moreover, mean RTs showed significant differences between emotionally positive and negative stimuli. For Valence Rating [*t*(3356) = −3.17, *p* < 0.001, *R*^2^ < 0.01], positive stimuli (*M* = 2348.77, *SD* = 1478.23) were, on average, rated faster than negative ones (*M* = 2559.69, *SD* = 1802.97). For Arousal Rating [*t*(3356) = 2.46, *p* = 0.01, *R*^2^ < 0.01], the opposite pattern was found (*M*_negative_ = 2398.98, *SD*_negative_ = 2128.59, *M*_positive_ = 2561.85, *SD*_positive_ = 1684.45).

Based on the optimal lambda suggested by the Box-Cox transformation test (λ = 0.46), Valence Rating was sqrt-transformed. The exclusion of extreme values as indicated by reaction times reduced data points by 0.06% (3,356 remaining data points). Following the stepwise elimination procedure (cf. Supplementary Material), the identified best-fitting model (*AIC* = 884.77, *BIC* = 927.60, log-likelihood = −435.39) included Valence Category, Stimulus Domain, and the interaction between both variables as fixed effects. The analysis yielded a statistically significant main effect of Valence Category [χ^2^(1,*N* = 3,356) = 1558.73, *p* < 0.001] but not Stimulus Domain [χ^2^(1,*N* = 3,356) = 0.25, *p* = 0.62]. On average, negative stimuli (*M* = 2.12, *SD* = 1.04) were rated more negatively than positive stimuli (*M* = 7.47, *SD* = 1.18).

**FIGURE 2:**
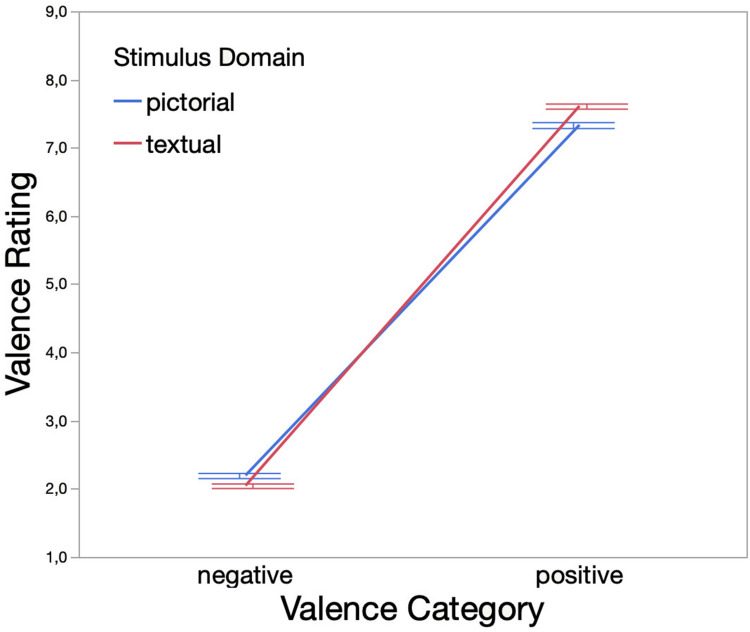
Interaction effect of Valence Category (i.e., negative, positive) and Stimulus Domain (i.e., pictorial, textual) on Valence Rating (assessed on a 9-point rating scale). Error bars denote one standard error from the mean. Descriptive statistics are as follows. Negative valence category: *M*_pictorial_ = 2.20, *SD*_pictorial_ = 1.11, *M*_textual_ = 2.05, *SD*_textual_ = 0.97; positive valence category: *M*_pictorial_ = 7.33, *SD*_pictorial_ = 1.27, *M*_textual_ = 7.61, *SD*_textual_ = 1.07.

Due to the significant interaction (see Figure 2) between Valence Category and Stimulus Domain [χ^2^(1,*N* = 3,356) = 29.41, *p* < 0.001], the main effect of Stimulus Domain was further explored within the subsets of positively and negatively valenced stimuli. The analysis indicated significant main effects of Stimulus Domain for both the negative [χ^2^(1,*N* = 1,677) = 11.49, *p* < 0.001] and positive stimuli [χ^2^(1,*N* = 1,679) = 33.43, *p* < 0.001]. Thus, emotionally positive vignettes (*M* = 7.61, *SD* = 1.07) were, on average, rated more positively than their pictorial counterparts (*M* = 7.33, *SD* = 1.27). The same superiority was observed within the negative valence category. Hence, emotionally negative vignettes (*M* = 2.05, *SD* = 0.97) were, on average, rated more negatively than emotionally negative pictures (*M* = 2.20, *SD* = 1.11).

With respect to ratings of Perceived Arousal, values were sqrt-transformed as indicated by the Box-Cox transformation test (λ = 0.71). Based on the reaction times, 13 extreme values (0.39%) were identified and subsequently excluded (3,345 remaining data points). The backward-reduction of fixed effects resulted in a random-intercepts model (*AIC* = 3462.4, *BIC* = 3492.9, log-likelihood = −1726.2) with Valence Category as sole predictor [χ^2^(1,*N* = 3,345) = 461.74, *p* < 0.001] indicating that emotionally negative stimuli (*M* = 6.44, *SD* = 1.87) were, on average, rated as more arousing than emotionally positive ones (*M* = 3.07, *SD* = 2.00).

### Eye Movements in Reading

**FIGURE 3:**
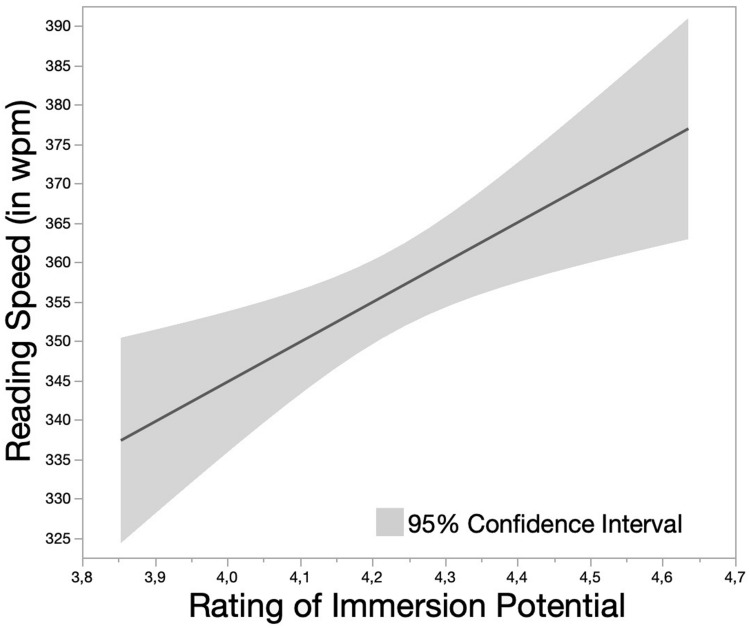
Main effect of Immersion Potential Rating on Reading Speed (in wpm). Immersion Potential was evaluated as one text characteristic in the pilot studies and rated on a 5-point rating scale.

Due to the rightward skewed distribution, Reading Speed was log-transformed (Box-Cox transformation test: λ = −0.14). The absolute criterion for the identification of extreme values was set to 1,000 wpm (exclusion of three data points). Fifty-nine further data points were excluded based on the relative criterion (in total: 3.69%; 1,616 remaining data points). Following the backward-elimination procedure, the best-fitting model (*AIC* = −1488.6, *BIC* = −1445.5, log-likelihood = 752.31) suggested significant main effects of Valence Rating [χ^2^(1,*N* = 1,616) = 4.36, *p* = 0.04], Arousal Rating [χ^2^(1,*N* = 1,616) = 3.85, *p* = 0.05], and Immersion Potential Rating [χ^2^(1,*N* = 1,616) = 4.44, *p* = 0.04]. The latter effect indicated a positive linear relationship between ratings of Immersion Potential and Reading Speed with faster reading in case of higher ratings on Immersion Potential (see Figure 3).

**FIGURE 4:**
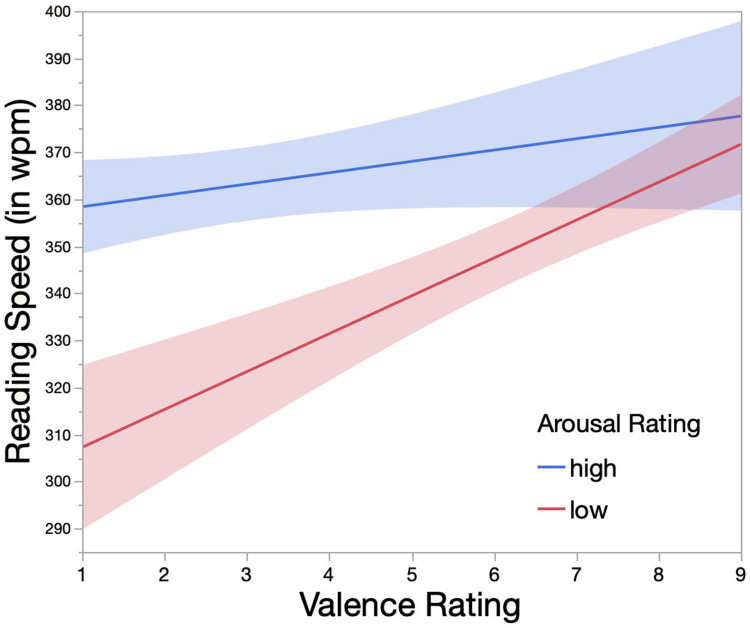
Interaction effect of Valence and Arousal Rating on Reading Speed (in wpm). Valence and Arousal Rating were evaluated on 9-point rating scales by participants of the eye tracking study. Arousal Rating was split into two factor levels (i.e., low versus high) using the quantcut-function from the gtools package (Warnes et al., 2018). Colored areas indicate the 95% confidence interval of each fitted line.

Since the interaction between Valence and Arousal Rating proved to be statistically significant [χ^2^(1,*N* = 1,616) = 7.84, *p* = 0.01], we further analyzed simple main effects by splitting the data based on Arousal Rating into two quantiles using the quantcut-function from the gtools package (Warnes et al., 2018). In this manner, the main effect of Valence Rating could be explored within two artificially constructed factor levels of Arousal Rating, one subset representing the low- (interval: [1–5]; *N* = 884) and the other the high-arousal group (interval: (5–9], *N* = 732). The main effect of Valence Rating reached statistical significance within the low- [χ^2^(1,*N* = 884) = 8.57, *p* = 0.003] but not high-arousal [χ^2^(1,*N* = 732) = 0.02, *p* = 0.89] subset (see Figure 4). In the low-arousal subset, positively valenced vignettes were, on average, read faster than negatively valenced ones. The main effect of Immersion Potential Rating remained significant within the high- [χ^2^(1,*N* = 732) = 4.44, *p* = 0.04] but not low-arousal group [χ^2^(1,*N* = 884) = 3.12, *p* = 0.08]. It should be noted that the results of the linear mixed-effects models within the two subsets have to be treated with caution. Since Valence and Arousal Rating were, in general, highly correlated, the artificially created arousal subsets possessed items disproportionally distributed over the valence categories, e.g., the high-arousal subset clearly contained more negatively than positively valenced vignettes.

For mean FFD, values were again transformed as indicated by the Box-Cox transformation test (λ = 1.43; 1/mean FFD). As absolute criterion, an upper threshold of 300 ms was applied (exclusion of one data point). Fifty-three further data points were excluded based on the relative criterion (in total: 3.22%; 1,624 remaining data points). Following stepwise model comparisons, the random-intercepts model with solely random effects was identified as best-fitting model (*AIC* = −21595, *BIC* = −21573, log-likelihood = 10802). Consequently, none of the considered predictors proved to be of explanatory value for mean FFD.

**FIGURE 5:**
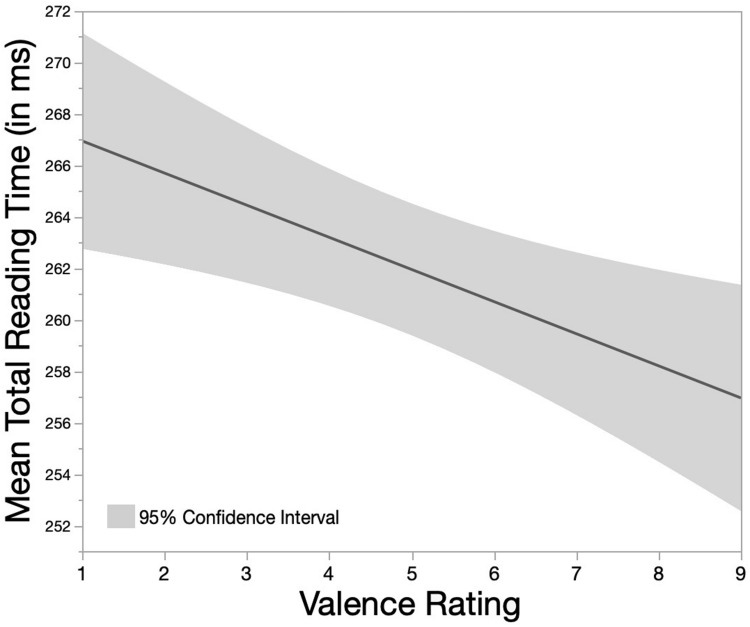
Main effect of Valence Rating on mean Total Reading Time (mean TRT in ms). Valence Rating was evaluated on a 9-point rating scale by participants of the eye tracking study.

With respect to mean TRT, a sqrt-transformation was applied due to the rightward skewed distribution (Box-Cox transformation test: λ = −0.46). Values over 500 ms were identified as extreme values and subsequently excluded (five data points). Based on the relative criterion, 62 additionally data points were removed (in total: 3.99%; 1,611 remaining data points). The backward-elimination procedure identified the intercepts-only model with Valence Rating as only predictor as best-fitting model (*AIC* = −13290, *BIC* = −13263, log-likelihood = 6650.0). The statistically significant main effect of Valence Rating [χ^2^(1,*N* = 1,611) = 6.05, *p* = 0.01] indicated that mean TRTs tended to decrease with increasing Valence Rating (see Figure 5).

### Eye Movements in Picture Viewing

As indicated by the Box-Cox transformation test (λ = 0.38), values for Mean Saccade Amplitude were sqrt-transformed. The absolute criterion for the exclusion of extreme values was set to 10° of visual angle (exclusion of four data points). Based on the relative criterion, 46 further data points were excluded (in total: 2.98%; 1,630 remaining data points). Values of Total Number of Fixations were squared as suggested by the Box-Cox transformation test (λ = 1.76). The upper threshold was set to a total number of 15 fixations (exclusion of five data points). Within the second step, 21 data points were additionally excluded (in total: 1.55%; 1,654 remaining data points). Lastly, Mean Fixation Duration was transformed due to the rightward skewed distribution (Box-Cox transformation test: λ = −1.23; 1/mean fixation duration). An upper limit of 800 ms was applied as absolute criterion for the identification of extreme values (exclusion of nine data points). Based on the relative criterion, 63 further data points were excluded (in total: 4.29%; 1,608 remaining data points).

For all three dependent variables, the intercepts-only models with solely random effects were identified as best-fitting models (for Mean Saccade Amplitude: *AIC* = −706.25, *BIC* = −684.66, log-likelihood = 357.12; for Total Number of Fixations: *AIC* = 15887, *BIC* = 15909, log-likelihood = −7939.5; for Mean Fixation Duration: *AIC* = −19644, *BIC* = −19622, log-likelihood = 9826.0). Hence, none of our predictors seemed to be suitable for the prediction of executed fixations and saccadic movements.

## Discussion

The aim of the present study was to examine effects of emotional content on subjective ratings of Perceived Valence and Arousal, eye movements in reading, and eye movements in picture viewing. With this aim, we asked a group of 42 participants to assess the emotional valence and arousal of 40 emotionally valenced (i.e., positive, negative) vignettes and pictures, respectively. To the best of our knowledge, this is the first study including a cross-domain comparison between more complex verbal materials (i.e., vignettes) and pictures providing matching semantic information.

As indicated by the reported descriptive statistics, the experimental manipulation of textual and pictorial valences proved to be successful. Lowest ratings of Perceived Valence were observed for negative, followed by neutral, and lastly positive stimuli. Furthermore, the wide range of individual ratings collected for each valence category stressed the necessity to go beyond the simplified categorical operationalization of emotional valence. Emotionally positive stimuli were, on average, rated as less arousing than emotionally negative stimuli. In line with previous studies on words (e.g., Võ et al., 2009; Soares et al., 2012; Söderholm et al., 2013; Montefinese et al., 2014), sentences (e.g., Pinheiro et al., 2017), and pictures (e.g., Verschuere et al., 2001; Lang et al., 2008; Dufey et al., 2011; Soares et al., 2015), the linear relationship between Perceived Valence and Arousal was found to be more pronounced for negatively compared to positively valenced stimuli. This observed asymmetry might possibly be due to the absence of erotic and thus high-arousal positive pictures in the NAPS database (cf. Marchewka et al., 2014). However, the strong correlation suggests that the two affective dimensions can rarely be studied apart from each other when focusing on effects of emotion induction in ecologically valid materials (cf. Citron, 2012).

With respect to the cross-domain comparison, vignettes attracted, on average, more extreme valence ratings than pictures supporting the assumed superiority of textual compared to pictorial materials. As expected, no effect of Stimulus Domain on Perceived Arousal was observed indicating that textual stimuli are able to induce arousal levels that are comparable to the ones elicited through pictures. Hence, the present study provides further evidence that verbal stimuli are at least as suitable for the induction of emotions as pictures. More specifically, the previously reported superior valence effects of emotionally positive words and phrases (e.g., Schlochtermeier et al., 2013; Tempel et al., 2013; Bayer and Schacht, 2014) applied not only to more complex linguistic material but also to negatively valenced ones. In contrast to Tempel et al. (2013), reaction times for valence ratings showed no significant differences between stimulus domains. Thus, while judgments of emotional valence required comparable processing times for both stimulus domains (cf. Schlochtermeier et al., 2013), vignettes attracted more extreme valence ratings than pictures.

Regarding emotion effects in reading, faster reading times for both vignettes perceived as emotionally positive (i.e., supralexical level) and their constituting content words (i.e., lexical level) were hypothesized. At the supralexical level, additional analyses exploring the significant interaction between Valence and Arousal Rating on Reading Speed revealed that the main effect of Perceived Valence applied to low-arousal vignettes, exclusively. Hence, vignettes rated as slightly arousing, emotionally positive were, on average, read faster than vignettes perceived as slightly arousing, emotionally negative. Reading speeds for high-arousal vignettes were found to be comparable to the one for low-arousal, emotionally positive vignettes independent of their perceived valences. Hence, vignettes perceived as emotionally negative required high arousal levels to show a similar processing advantage as vignettes perceived as emotionally positive. The observed effect conforms to previous findings from lexical decision tasks showing that the processing of emotionally negative, but not positive, words depends on their arousal levels. More specifically, reactions to negatively valenced words were reported to be slower for low- versus high-arousal stimuli (e.g., Nakic et al., 2006; Hofmann et al., 2009; Recio et al., 2014). In other words, in line with studies examining reaction latencies, valence effects on Reading Speed were absent for vignettes perceived as highly arousing.

To our knowledge, there is only one further study primarily focusing on effects of textual valence on eye movements during reading of narratives. In accordance with our results, Ballenghein et al. (2019) were able to find shortest reading times for emotionally positive texts. However, statistically significant differences were restricted to the comparison of mean fixation durations between emotionally positive and neutral narratives. Hence, the present study extends their findings by revealing statistically significant differences between positively and negatively valenced vignettes when using individual ratings instead of valence categories. Interestingly, Ballenghein et al. (2019) likewise observed that reading in emotionally negative texts is influenced by arousal. More specifically, the authors reported significantly shorter mean fixation durations for high-arousal, emotionally negative texts compared to their medium-arousal counterparts.

In accordance with the NCPM (Jacobs, 2011, 2015b), vignettes associated with higher ratings of Immersion Potential attracted, on average, faster reading. The multi-dimensional phenomenon of immersive reading is related to various factors including characteristics of the text (e.g., easy-to-recognize words; Jacobs, 2015b), the context (e.g., action-oriented descriptions; Kuijpers et al., 2014), and the reader (e.g., identification with the protagonist; Jacobs, 2015b). Since the vignettes were constructed to be easily understandable, to emotionally engage the reader, to enhance the identification with the protagonist, and likely activated familiar situation models as their contents were based on pictures of daily situations, the overall high ratings on Immersion Potential are not surprising. Hence, it has to be considered that the reported effect of Immersion Potential is based on a comparably low value range (i.e., range: 3.89–4.6 on a 5-point rating scale). Immersion Potential was neither explicitly manipulated nor systematically measured with commonly used scales such as the Story World Absorption Scale (Kuijpers et al., 2014). Thus, while the reported significant main effect of Immersion Potential is in line with the assumptions of the NCPM, the effect needs to be replicated by materials especially constructed or selected to study effects of immersion.

The effect of Perceived Valence reported at the supralexical level could also be observed at the lexical one. As expected, content words of vignettes perceived as emotionally positive attracted, on average, faster reading as indicated by shorter mean TRTs. However, effects of (perceived) emotional valence were missing for mean FFD suggesting an absence of valence-specific effects at early processing stages. Comparable findings are provided by EEG studies examining the time course of emotional word processing (Citron, 2012, for review). In this regard, effects of emotional content have been shown to appear at early and later processing stages (e.g., Herbert et al., 2006, 2008; Kissler et al., 2007; Hofmann et al., 2009; Schacht and Sommer, 2009b; Bayer et al., 2010). Whereas the early effect has been assumed to be predominantly governed by arousal, valence-driven modulations have been put forward in explanations of the later impact indicating a deeper encoding of positive stimuli (e.g., Delplanque et al., 2004; Herbert et al., 2006, 2008; Kiefer et al., 2006; Conroy and Polich, 2007; Kissler et al., 2009). Whether the here reported shorter total reading times for as positive perceived vignettes and their constituting content words are in line with the assumed deeper encoding for positive compared to negative words is still an open question for future empirical research. So far, our results conform to the positivity bias during meaning construction (cf. Jacobs et al., 2015; Lüdtke and Jacobs, 2015) assuming an easier semantic integration and construction of situation models for verbal materials including emotionally positive compared to negative words. Further studies have to be conducted to explore under which circumstances such processing advantage may cause deeper encoding.

With respect to eye movements in picture viewing, descriptive statistics revealed that emotionally negative pictures tended to attract slightly shorter fixations and longer saccades compared to emotionally positive ones. However, neither Perceived Valence nor Arousal were of explanatory value for the three examined eye tracking parameters. Hence, emotion effects were absent for both fixation (i.e., Mean Fixation Duration, Total Number of Fixations) and scan (i.e., Mean Saccade Amplitude) patterns as hypothesized and previously reported by studies with a comparable experimental design (Bradley et al., 2011; Niu et al., 2012).

### Limitations and Future Directions

Building on the lastly mentioned pictorial materials, results of the present study have to be interpreted within the framework of the particular viewing paradigm and performed analysis. Hence, it remains an open question whether valence-specific effects would be present when analyzing certain areas of interest (e.g., focal object versus background; Yang et al., 2012) or focusing on temporal dynamics in picture viewing. With respect to the latter, differences between positive and negative valences have been reported when emotional and neutral pictures were presented at the same time competing for attentional resources (e.g., Calvo and Avero, 2005; Simola et al., 2013).

Complementary to studies focusing on single word processing (e.g., Kousta et al., 2009; Briesemeister et al., 2011; Lüdtke and Jacobs, 2015), (perceived) positive valence showed comparable facilitative effects when manipulated at the text level. This hypothesized effect conforms to the aforementioned high correlation between the text and lexical levels (Bestgen, 1994; Whissell, 2003; Hsu et al., 2015b; Jacobs, 2015b). In general, the present distribution of Reading Speed in wpm indicated that our participants read rather fast (*M* = 367.48, *SD* = 123.97), with an average of 330 wpm being considered as a threshold for fast reading (cf. Rayner et al., 2010). This observation emphasizes the low cognitive demands and high comprehensibility of our linguistic materials (cf. Cupchik et al., 1998; Søvik et al., 2000; Rayner et al., 2006; Liversedge et al., 2011; Lüdtke et al., 2019).

In this context, the influential role of our evaluative judgment task ought to be considered. Hence, both the comparable low task demands (e.g., no comprehension questions) and the strong focus on clearly emotionally valenced materials (i.e., no neutral stimuli) might have influenced participants’ reactions and compliance with the task (Westermann et al., 1996; Estes and Verges, 2008). Although it might have been possible that participants performed the task without having read the vignettes entirely, visual inspections of fixation patterns indicated that they did not stop reading after the first sentences. Apart from the fact that evaluative judgment tasks have largely been applied in the context of emotion induction (e.g., Schupp et al., 1997; Bradley et al., 2001; Calvo and Lang, 2004; Nummenmaa et al., 2006; Brunyé et al., 2011; Schlochtermeier et al., 2013; Mouw et al., 2019; Child et al., 2020), effects of emotion have likewise been reported for tasks not explicitly focusing on the affective content (e.g., Schacht and Sommer, 2009a; Rellecke et al., 2011; Lüdtke and Jacobs, 2015). Consequently, it has been shown that encoding of emotional valence takes place even when affective processing is not necessary to perform the task. Nevertheless, up to which level the here reported effects of perceived emotional valence could be observed in other reading situations remains an open empirical question.

Since the focus of the present study was on the induction of emotions in a cross-domain comparison, neutral stimuli were largely neglected. Consequently, future research has to reveal whether the reported effects of Perceived Valence remain stable when including neutral materials. However, it should be noted that emotional valence is highly prevalent in objects of everyday life making it difficult to select appropriate neutral stimuli (cf. Lebrecht et al., 2012). With respect to the comparison between vignettes and pictures, the slightly different presentation mode has to be considered. Whereas vignettes required self-paced reading, pictures were presented within a commonly used 3-s time interval (e.g., Calvo and Lang, 2004; Calvo and Avero, 2005; Nummenmaa et al., 2006; Yang et al., 2012; Bayer and Schacht, 2014; Marchewka et al., 2014). However, we are convinced that the reported domain-specific effects are stronger related to differences associated with the processing of pictures compared to vignettes than to different presentation modes.

As a step toward the use of more ecologically valid stimuli in psychological reading research, reading behavior was examined in self-constructed vignettes. Although they do not represent natural reading materials such as excerpts of well-known books (e.g., Hsu et al., 2014, 2015a,b, 2015c), their major advantage concerns the opportunity to easily and systematically control or rather manipulate various variables of interest (e.g., choice of words, number of protagonists). The thus acquired results can consequently be used to inform about potentially influential covariates that ought to be considered in future studies.

As mentioned in the introduction, the simplest model about the valence of supralexical units like vignettes would assume that the global valence is a (linear or non-linear) function of the valence values of its constituents. Following this idea, mean TRT and mean FFD were computed by averaging the fixation durations of all content words constituting a vignette. Consequently, lexical word features such as length, frequency, word position and repetition were neglected (Raney et al., 2000; Kuperman et al., 2010, for reviews). Moreover, since the present study aimed to investigate natural reading processes, our study material could only be controlled for a limited set of variables. However, current computational quantitative narrative and sentiment analysis tools such as “QNArt” and “SentiArt” (Jacobs, 2018a,b,^2019^) provide the possibility of quantitatively describing words on a wide range of lexical affective-semantic features. To investigate their possibly interactive impact on eye movements in reading, other approaches than the applied linear mixed-effects models are of inevitable necessity.

In this context, machine learning assisted methods of predictive modeling offer a promising and valuable alternative or complementary perspective (e.g., Yarkoni and Westfall, 2017; Vijayakumar and Cheung, 2018). Since the approach allows working with many intercorrelated variables and non-linear data patterns (Goodman et al., 2016; Cheung and Jak, 2018), it is particularly well suited for analyzing effects of literary texts on reading behavior (Jacobs, 2018a,b; Xue et al., 2019). The combined application of QNA data and machine learning algorithms such as neural networks or decision trees has yielded promising results in previous research on the beauty of words (Jacobs, 2017), the literariness of metaphors (Jacobs and Kinder, 2017, 2018), or the comprehensibility and emotion potential of poetic texts (Jacobs and Lüdtke, 2017; Jacobs et al., 2017; Jacobs, 2018a,b; Xue et al., 2019). Future research will have to provide comparable analyses for reading in prose (e.g., vignettes).

## Conclusion

Considering that our results indicated that emotional vignettes are able to induce stronger valence effects than their pictorial counterparts, it can be proposed that the present study provides further evidence for the suitability of textual materials in the area of emotion induction. Furthermore, this is the first eye tracking study showing a statistically significant difference in effects of positive and negative, and not only of emotional and neutral texts. In this context, results from previous experiments using isolated words and sentences could be replicated at the supralexical level: perceived positive text valence attracts shorter reading times than perceived negative valence at both the supralexical and lexical level.

## Data Availability Statement

The datasets generated for this study are available on request to the corresponding author.

## Ethics Statement

The studies involving human participants were reviewed and approved by the Ethics Committee of the Department of Education and Psychology at Freie Universität Berlin. The patients/participants provided their written informed consent to participate in this study.

## Author Contributions

FU and JL contributed to the conception and design of the study. FU developed the test stimuli and collected the data. FU performed the statistical analysis in consultation with AJ and JL. FU wrote the first draft of the manuscript. All authors contributed to the manuscript revision, read and approved the submitted version.

## Conflict of Interest

The authors declare that the research was conducted in the absence of any commercial or financial relationships that could be construed as a potential conflict of interest.

## Footnotes

1 http://www.sr-research.com/experiment-builder

2 Normative ratings of valence were originally collected on a continuous sliding scale ranging from 1 (very negative) to 9 (very positive), with 5 indicating a neutral valence.

3 http://www.soscisurvey.de/

4 Based on ratings from 13 participants, the following statistics can be reported for neutral vignettes: Valence: *M* = 4.85, *SD* = 0.93; Arousal: *M* = 4.79, *SD* = 0.89; Comprehensibility: *M* = 4.77, *SD* = 0.54; Immersion Potential: *M* = 3.64, *SD* = 1.16; Emotion Induction Potential: *M* = 2.46, *SD* = 1.14.

5 Previous studies reported effects of participants’ mood on eye movements in both picture viewing (Wadlinger and Isaacowitz, 2006) and reading (Scrimin and Mason, 2015).

6 http://www.sr-research.com/data-viewer/

7 https://www.jmp.com/de_de/home.html

8 https://cran.r-project.org/
