# Supplementary material for: From Abstract Symbols to Emotional (In-)Sights: An Eye Tracking Study on the Effects of Emotional Vignettes and Pictures
Source: Front Psychol. 2020 May 26;11:905. doi: 10.3389/fpsyg.2020.00905 (PMC7264705; doi:10.3389/fpsyg.2020.00905)
Supplement: Supplementary file 1 [file Table_1.pdf]

Supplementary Material

1 Emotionally negative vignettes

Table S1

Emotionally negative vignettes and their corresponding NAPS picture IDs

Picture ID[1]	Vignette	Number of words	Mean valence rating (1-9)[2]
Animals_013_h	Es ist ein kalter Wintermorgen und du bist auf deiner Lieblingsstrecke im Wald spazieren. Plötzlich hörst du einen lauten Knall – durch die Bäume siehst du ein Auto, das sich schnell auf dem nahen Feldweg entfernt. Du läufst schnell zu der Stelle des Aufpralls. Als du dich näherst, siehst du etwas Braunes im Schnee des Straßengrabens liegen. Es ist ein kleines süßes Rehkitz. Sein Körper liegt verdreht auf der Seite, der Kopf ist nach hinten überstreckt und seine Augen schauen weit offen und dunkel ins Leere.	85	2.96
Animals_024_h	Du arbeitest schon länger als Aushilfe in einem Restaurant in der Nähe. Deine Chefin schickt dich ins Lager, um weitere Servietten und Kerzen zu holen. Als du ins Lager kommst, hörst du klägliche, röchelnde Laute. Glasscherben liegen auf dem Boden. Ein schwarzes Katzenbaby mit blutiger Schnauze und einer tiefen Schnittwunde an seinem kleinen Kopf liegt vor dir. Es muss kopfüber durch das Glas gebrochen sein, denn das Fleisch an seiner Nase ist bis auf den Knochen abgeschabt. In einem seiner Augen befinden sich ebenfalls Glasscherben.	85	2.02
Animals_039_h	Es müssen eine Menge Nägel gewesen sein, die der große schwarzbraune Terrier gefressen hat. Du hoffst, dass du und deine Kolleg*innen ihn noch retten können. Ihr habt das sich vor Schmerzen windende Tier auf den OP-Tisch gelegt und ihm eine Narkose-Spritze gegeben. Der Tropf ist gelegt, und der bewusstlose Hund ist trotz Narkose auf dem OP-Tisch festgebunden. Du öffnest langsam mit deinem Skalpell die Bauchdecke des Hundes. Die entstandene Öffnung befestigst du mit den Klammern, greifst mit deinen Händen in den Bauchraum und hebst vorsichtig den Darm heraus.	88	2.89

Animals_054_h	Du hast selten einen so vernachlässigten Hund gesehen. Die dünne Haut schlottert ihm um die Knochen, er muss unvorstellbaren Hunger gehabt haben. Sein Fell ist stumpf und ungepflegt, er riecht nach Kot und altem Urin. Er muss Schmerzen haben, wenn er läuft. Seine Nägel sind so lang, dass sie zu allen Seiten abstehen und ihm von unten und von der Seite ins Fleisch schneiden. Gebückt steht er da, mit gesenktem Kopf. Seine dunklen Augen sind leer. Er hat jede Hoffnung auf ein würdiges Leben verloren.	85	2.38
Animals_063_h	Das kleine Rehkitz liegt wie versteinert in der blutverschmierten Plastikplane vor dir. Seine Augen sind vor Angst und Panik weit aufgerissen und blicken unbeweglich auf den Boden. Ein Wolf hat seine Mutter angefallen und es dabei verletzt. Sein Hinterbein ist aufgerissen und der Angreifer hat eine große, klaffende Wunde hinterlassen. Es sieht aus, als wäre dem kleinen Rehkitz ein Stück Fell abgezogen worden. Der Wolf hat ihm ein großes Stück Fleisch aus dem Hinterbein gerissen, ehe er es liegen gelassen hat, um die Mutter des Rehkitzes zu töten.	88	2.33
Animals_077_h	Je näher du den Käfigen kommst, desto stärker stinkt es nach Kot und Urin. Viele der Hunde haben Wunden, die schlecht oder gar nicht verheilt sind und sich in dem Dreck, in dem sie leben müssen, entzündet haben. Etliche kratzen sich oder beißen sich die schlecht geheilten Wunden wieder auf. Der Hündin im ersten Käfig steht das Fell vor Dreck in alle Richtungen ab. Sie hat eine klaffende Wunde an der Kopfseite, die schon älter sein muss. Unter der herabhängenden Haut siehst du die entzündete Wunde.	86	2.22
Faces_009_h	Du blickst in das Gesicht eines Kindes. Seine Haut ist bleich, fast totengleich. Sie sieht aus, als wäre sie aus Wachs. Das Gesicht des Kindes ist übersäht mit Schnittwunden. In seinen geweiteten Nasenlöchern ist getrocknetes Blut. Zwischen Oberlippe und Nase hat das Kind eine Hasenscharte, durch die du die Zähne und das Zahnfleisch sehen kannst. Seine Augen schauen nach oben, es schielt etwas. Das Kind blickt ohne Erwartungen und ohne Angst in die Kamera. Der Schnitt auf seiner Stirn ist tiefer als die anderen. Die dunkelrote, klaffende Wunde leuchtet auf der weißen, bleichen Haut.	94	2.13

Faces_146_h	Auf deinem allmorgendlichen Rundgang auf der Intensivstation betrittst du das erste Zimmer. Dort liegt ein Mann mit weit geöffneten Augen seitlich auf seinem Bett. Er blickt vollkommen starr nach oben ins Leere. Um dich herum ist alles ruhig. Du bemerkst, dass die Maschinen abgeschaltet worden sind. Der Beatmungsschlauch liegt noch neben seinem geöffneten Mund auf der Matratze. Seine Lippen sind blutleer. Du kannst die geplatzten Äderchen in den Augen erkennen. Er sieht müde und abgekämpft aus. Eine gewisse Ungläubigkeit liegt in seinen Augen. Sein Mund ist zu einem endlosen, schmerzhaften Stöhnen geöffnet.	92	2.46
Faces_152_h	Du siehst eine Frau nach einer Hauttransplantation im Gesicht. Es ist eine junge Frau, fast noch ein Kind. Die brennende Säure hat ihr Gesicht verätzt. Man sieht die Spuren der Transplantation, die Vernarbungen ihrer Haut. Die Augen und der Mund haben einen gequälten Ausdruck. Das rechte Ohr und ein Teil des Gesichtes sind von einem Mullverband bedeckt. Zum Schutz vor Blicken und der Sonne hat sie das Kopftuch wie einen Schirm etwas über die Stirn gezogen. Ihre großen, dunklen Augen stehen in starkem Kontrast zu ihren Verletzungen.	87	3.20
Faces_159_h	Du machst deine Runde auf der Kinderintensivstation und siehst eine neue Patientin. Die Haut des Kindes ist im Gesicht verbrannt. Die verklebten Augen sind geschlossen. Das linke Auge ist halb geöffnet, der Blick ist leer. Du fragst dich, ob das Kind begreifen wird, was geschehen ist. Die Lippen sind bis zur Unkenntlichkeit aufgequollen, der Mund ist leicht geöffnet. Eine weißliche Cremeschicht bedeckt das Gesicht und die Augen, nur die Nasenlöcher sind frei. Die Haut darunter ist gerötet. Das Gesicht wirkt wie versteinert, apathisch. Es sieht mehr tot als lebendig aus.	90	2.02
Faces_172_h	Unter den alten Decken liegt ein abgemagerter, alter Mann. Du siehst, dass seine Haut wie eine dünne Papierschicht über seine abstehenden Knochen gespannt ist. Sein dürrer Körper ist von der Krankheit geschwächt. Sie scheint ihn von innen aufzufressen. In seinem leicht geöffneten Mund siehst du seine letzten verfaulten Zahnstummel. Jede Rippe drückt sich durch seine Haut. Er sieht aus wie ein lebendiges Skelett. Seine dünnen Arme sehen	88	2.60

	zerbrechlich aus, seine Augen starren wie blind ins Leere. Eine Fliege krabbelt über sein Kopfkissen. Er sieht trostlos und hoffnungslos aus.		
Faces_283_h	Eine alte Frau steht schluchzend vor dir, sie hat ihre Hände wehklagend gen Himmel erhoben. Ihre Augen sind geschlossen, als wollte sie nicht wahrhaben, was gerade geschieht. Das Gesicht ist vor seelischer Qual verzogen. Tiefe Sorgenfalten ziehen sich durch ihr Gesicht. Hinter ihr lodern die unerbittlichen Flammen aus ihrem Zuhause. In einem Haus quillt aus den Fenstern dichter pechschwarzer Rauch. Die Fenster sind zerborsten, Trümmer liegen auf der Straße. Ihre Mimik zeigt die unermessliche Trauer und den Schmerz, der sie verzerrt wie ein loderndes Feuer.	85	2.13
Faces_284_h	Der Mann ist mit einer Jacke bekleidet, deren weiße Farbe du unter dem vielen Dreck und Ruß kaum sehen kannst. Du siehst, dass seine beiden Beine amputiert worden sind. Das eine Bein ist so kurz amputiert worden, dass du es nicht sehen kannst. Das andere ist noch bis zur Hälfte des Schienbeins vorhanden. Mit seinem nackten, amputierten Bein sitzt der Mann auf dem harten Asphalt und hebt beide Arme. Sein Gesicht ist dreckig, seine Augen sind zusammengekniffen. Neben ihm liegt Müll. Er raucht eine selbstgedrehte Zigarette.	86	3.18
Faces_290_h	Du stehst vor einem alten, verwahrlosten Mann, der auf der Bettkante in einem kleinen Häuschen sitzt. Das Haus ist verfallen und zugemüllt. Die Schultern des Mannes fallen nach vorne und sein Kopf ist gesenkt. Der dichte, dreckige Bart verdeckt sein halbes Gesicht und seinen Hals. Es sieht aus, als wäre auch getrocknetes Blut auf seinem Bart. Über seinen kleinen Augen liegt eine tiefe Falte auf der Stirn. Eine große, tiefe Traurigkeit blickt aus seinen Augen. Er sieht aus, als habe er keine Perspektive mehr. Als hätte er seine Träume schon lange aufgegeben.	92	3.02
Faces_293_h	Ein Mann wird bäuchlings von vier Polizisten weggetragen. Sein Mund ist zu einer schmerzerfüllten Grimasse verzogen. Seine Augen sehen nach hinten, wo seine Arme schmerzhaft auf seinem Rücken verdreht sind. An der verdrehten Armen halten ihn zwei der Polizisten fest. Du fragst dich, was er getan hat, dass die Polizisten ihn so	89	2.71

	brutal behandeln. Die Stirn des Mannes liegt in Falten und seine Augen sind weit aufgerissen. In seinen Augen liegen die Überraschung und Wut über die Festnahme. Die Muskeln seines Gesichts und seines Halses sind vor Schmerz angespannt.		
Faces_298_h	Du hast unterschätzt, wie blutig dieser Sport ist. Du sitzt zitternd in der ersten Reihe und kannst das verletzte Gesicht des Kampfsportlers aus nächster Nähe sehen. Das Blut ist bis auf seinen kompletten, nackten Oberkörper gespritzt. Am Nasenrücken ist er am schlimmsten getroffen und er blutet stark aus der Nase. In seinem Mund siehst du das getrocknete Blut der ersten Runde. Er sieht müde und abgekämpft aus. In seinen Augen siehst du die geplatzten Äderchen. Seine Erschöpfung steht ihm ins Gesicht geschrieben. Er scheint am Ende seiner Kräfte und seiner Karriere zu sein.	93	3.16
Objects_003_h	Durch den Aufprall waren die beiden Autos weit voneinander weggeschleudert worden. Diesen Wagen hatte es besonders schlimm getroffen, er war komplett zerstört worden. Du denkst an die Insassen. Sie können den Unfall unmöglich überlebt haben. Fahrer- und Beifahrersitz waren durch den Zusammenstoß so ineinandergeschoben worden, dass kaum mehr zu erkennen war, wie das Auto einmal ausgesehen haben mochte. Das Dach hatte sich ebenfalls abgelöst und war wie eine Ziehharmonika zusammengedrückt worden. Falls jemand hinten auf den Sitzen gesessen hatte, musste diese Person mindestens stark verletzt worden sein.	87	2.76
Objects_139_h	Beim Spazieren bleibst du erstarrt stehen. Auf dem Weg liegt ein Kopf, zerkratzt und schmutzig, die kurzen, schwarzen Haare zerzaust. Ein langer Riss klafft von der Schläfe bis zum Kinn. Die schwarzen Augenhöhlen starren ins Leere. Beim zweiten Blick siehst du, dass es kein echter Kopf ist, sondern der einer Plastikpuppe. Die einzelnen Strähnen der Haare sind bis ins Kopfinnere eingedrückt. Das Innere des hohlen Kopfes und das Gesicht sind mit Erde verschmiert. Die Puppe eines Kindes wurde anscheinend brutal auseinandergerissen und der Kopf zertrümmert. Die Augen muss der Täter ebenso herausgerissen haben.	93	2.40
People_001_h	Sie steht vor dir in ihrer verbrannten Küche und schlägt die Hände vor das Gesicht. Es wirkt auf dich, als wollte sie nicht sehen, was sie alles verloren hat.	93	2.73

	Vielleicht will sie auch ihre Tränen verbergen. Du fühlst mit ihr die tiefe Verzweiflung, die ein Loch in ihre Seele gebrannt hat. Sie scheint immer noch in ihrem Bauch zu lodern. Du hörst an ihrem Wehklagen, dass sie nicht weiß, wie sie das ertragen soll. Alles scheint für sie bedeutungslos geworden zu sein. Ihr Gesicht und ihr Körper müssen sich leer und taub anfühlen.		
People_127_h	Du beobachtest zwei junge Männer, die einen Mann schlagen, der auf einer Wiese am Boden liegt. Der eine benutzt dafür einen Stock und der andere einen Schlagring, das heißt, sie sind schon bewaffnet unterwegs gewesen. Zwei andere Männer sind dabei und schauen zu. Es ist taghell und der Mann liegt am Boden und hält sich die beiden Arme schützend über den Kopf. Er krümmt sich vor Schmerzen und hat Angst um sein Leben. Der Mann mit dem Schlagring holt weit aus, um den Kopf des Opfers mit voller Wucht zu treffen.	91	1.36

Notes. Vignettes were constructed based on pictures from the *Nencki Affective Picture System* (NAPS; Marchewka et al., 2014).

[1] Picture ID refers to the corresponding ID from the NAPS.

[2] Further information regarding the online pilot studies can be requested from the 1st author.
