# Supplementary material for: From Abstract Symbols to Emotional (In-)Sights: An Eye Tracking Study on the Effects of Emotional Vignettes and Pictures
Source: Front Psychol. 2020 May 26;11:905. doi: 10.3389/fpsyg.2020.00905 (PMC7264705; doi:10.3389/fpsyg.2020.00905)
Supplement: Supplementary file 2 [file Table_2.pdf]

Supplementary Material

2 Emotionally positive vignettes

Table S2

Emotionally positive vignettes and their corresponding NAPS picture IDs

Picture ID[1]	Vignette	Number of words	Mean valence rating (1-9)[2]
Animals_131_h	Heute ist ein schöner Frühlingstag. Es hat aufgehört zu regnen, die Sonne scheint und der Himmel klart auf. Motiviert entschließt du dich, draußen entlang am stillen See spazieren zu gehen. Nach einigen Minuten bleibst du überrascht stehen: Nur wenige Meter von dir entfernt entdeckst du eine weißbraune Ente mit ihren kleinen Küken. Sie sind gerade dabei vom Land zurück ins Wasser zu gehen. Gefesselt beobachtest du, wie all die kleinen Küken eifrig ihrer Mutter folgen. Gern würdest du noch näher heran, um die weichen Federn zu berühren, doch du willst die Enten-Familie nicht erschrecken.	94	7.00
Animals_137_h	Du verbringst deine freie Woche in einem marokkanischen Urlaubsort. Du hast diese Reise schon lange geplant und mit großer Vorfreude auf deinen ersten Urlaubstag hingearbeitet. Gespannt auf all die neuen Erfahrungen hast du für heute eine Kamel-Wüsten-Tour gebucht. Als du am Treffpunkt ankommst, stehen die Kamele bereits gesattelt vor dir. Du betrachtest das weiche, hellbraune Fell und gehst immer näher heran. In der Ferne siehst du grüne Palmen und den hellblauen Himmel. Fasziniert von diesen Eindrücken kannst du es kaum noch erwarten, zum ersten Mal auf einem Kamel zu reiten. Das wird ein unvergessliches Erlebnis!	95	6.60
Animals_163_h	Du nutzt den wunderschönen Sommertag, um gemeinsam mit deinen Freunden in den örtlichen Tierpark zu gehen. Dort angekommen fesselt eines der zahlreichen Tiere deine Aufmerksamkeit: Es ist ein junges, schwarzweiß geflecktes Kamel. Es ist kaum größer als du und steht direkt vor dir. Du wagst dich näher heran und kannst es sanft berühren. Du kannst seine großen, kugelrunden Augen und seine kleine, schwarze Nase ganz genau sehen. Die Mundwinkel sind leicht angehoben, sodass du glaubst, das Kamel lächelt dich an. Du streichelst	92	6.77

	sein weiches Fell und willst am liebsten nicht mehr weitergehen.		
Animals_167_h	Du bist seit langem wieder einmal zu Hause und verbringst den Tag draußen im Garten. Als du dich umschaust, entdeckst du zu deiner Freude vor einem der Bäume eure orange-weiß gestreifte Katze. Mit ruhigem Schritt gehst du näher heran und hockst nun direkt vor ihr. Sie bleibt gelassen sitzen und blickt weiterhin zur Seite. Anscheinend beobachtet sie etwas in der Ferne. Du kannst jedes einzelne weiße Schnurrhaar ganz genau erkennen. Deine Anwesenheit scheint sie nicht zu ängstigen. Im Gegenteil: Ihr seid euch nur allzu gut vertraut.	86	6.96
Animals_172_h	Du verbringst den Tag zusammen mit deiner Familie im Zoo. Auf einer der grünen Wiesen breitet ihr eure Decke aus, um euch ein wenig auszuruhen. Als du nach links blickst, entdeckst du überrascht einen wunderschönen Pfau. Leise und ruhig gehst du interessiert bis auf einem Meter heran. Auf einmal breitet er seine bunten Federn zu einem prachtvollen Halbkreis aus. So etwas Faszinierendes hast du lange nicht mehr gesehen! All die leuchtenden und harmonischen Farben! Der Pfau bleibt selbstsicher und zugewandt vor dir stehen. Du nutzt den einzigartigen Augenblick, um dir seine beeindruckenden Federn genau anzuschauen.	95	6.67
Animals_191_h	Du bist zu Besuch bei deinem besten Freund. Da er dir unbedingt etwas zeigen möchte, zieht er dich raus in den Garten. Du folgst ihm neugierig und vollkommen ahnungslos. Abrupt bleibt er stehen und du blickst auf den Boden. Da sitzt es: ein kleines Katzenbaby, kaum größer als deine beiden Hände. Zutraulich geht es langsam auf dich zu. Sein Fell glänzt in den Farben Schwarz, Weiß und Braun. Auch seine rosafarbene Nase fällt dir sofort auf. Mit seinen großen Augen schaut es dich gespannt an und du möchtest es am liebsten auf deinen Arm nehmen.	95	7.58
Faces_066_h	Du bist zu Besuch bei deiner besten Freundin. Im Eingangsbereich bleibst du kurz stehen. Vor dir läuft ihr dreijähriger Sohn mit dem Staubsauger in der Hand über die Fliesen. Er schaut auf den Boden und wirkt vollkommen konzentriert, als ob die Aufgabe im Moment wirklich das Wichtigste für ihn sei. Er blickt noch nicht einmal zu dir auf. Es scheint so, als ob er mit seiner Arbeit in vollem Umfang zufrieden	96	6.62

	sei. Du gewinnst den Eindruck, dass er von seinen Hausarbeitsfähigkeiten völlig überzeugt ist. Sein Gesichtsausdruck spiegelt dein Gefühl gänzlich wider. Er wirkt selbstbewusst und zufrieden.		
Faces_079_h	Vor dir siehst du eine junge, schwarzhaarige Frau. Sie liegt lächelnd neben ihrem nur wenige Tage alten Kind. Es ist von einer hellgrünen Decke umgeben und schläft zufrieden und ruhig in ihrem Arm. Am strahlenden Gesicht der Mutter kannst du erkennen, dass sie ausnahmslos glücklich ist. Neben ihr scheint das Wichtigste in ihrem Leben zu liegen. Du siehst ihr die Strapazen der Geburt überhaupt nicht mehr an. Im Gegenteil: Sie wirkt entspannt und dankbar. Ihr breites Grinsen verrät dir, wie erfreut und sorgenfrei sie in diesem Moment ist.	88	7.47
Faces_100_h	Du schaust dir die Urlaubsbilder vom letzten Sommer an. Eines der Bilder betrachtest du besonders lange. Du erinnerst dich noch sehr gut an den Moment der Aufnahme: Auf dem Foto sind zwei kleine Brüder zu sehen. Sie stehen nebeneinander. Deine Aufmerksamkeit richtet sich vor allem auf ihre Gesichtsausdrücke. Beide grinsen über beide Ohren, sodass du ihre weißen Zähne sehen kannst. Ihre Augen strahlen und du kannst eindeutig erkennen, wie erfreut sie in dieser Situation sind. Ihre Mimik weckt ein Gefühl der Herzlichkeit und des Glücks in dir.	87	6.73
Faces_104_h	Es ist Sommer und du nutzt deine Pause, um im Stadtpark ein wenig zu entspannen. Du schaust dich um und entdeckst in der Nähe ein kleines Mädchen, das in die Richtung ihrer Mutter schaut. Ihr ehrliches Lächeln fesselt deine Aufmerksamkeit. Ihr Mund ist zu einem breiten Grinsen geformt, ihre leicht geröteten Wangen sind angehoben und ihre kleinen Augen strahlen vor lauter Freude. Ihr Gesichtsausdruck wirkt vollkommen natürlich und belebend. Sie scheint die gemeinsame Zeit mit ihrer Mutter sehr zu genießen und läuft weiter in ihre Richtung.	86	7.24
Faces_107_h	Gerade läufst du über eine grüne Parkanlage. Als du nach rechts schaust, musst du schmunzeln: Dort liegt ein kleiner Junge neben seinem Vater und rollt sich lachend auf dem Boden. Er hat den Mund weit offen und kann nicht mehr aufhören zu grinsen. Seine Augen sind geschlossen und er hält sich bereits den Bauch vor lauter Lachen. Er strahlt über beide	95	7.51

	Ohren. Sein Grinsen ist mitreißend und hervorstechend. Auch sein Vater kann seine Freude nicht mehr verbergen. Er blickt seinen Sohn begeistert an. Sein Lächeln ist ehrlich und authentisch. Die beiden strahlen pure Lebensfreude aus.		
Faces_108_h	Du bist mit deinem jüngeren Bruder an einer kleinen Wasseranlage. Dein Bruder nutzt die Gelegenheit und springt ins Wasser. Du beobachtest ihn, wie er zufrieden und glücklich an einem Wasserstrahl sitzt. Er hat seine Augen geschlossen und grinst breit. Seine Haare sind schon komplett nass. Du erfreust dich an seinem bezaubernden Lächeln. Er scheint den Moment vollkommen zu genießen und vergnügt sich am erfrischenden Wasser. Sein Lächeln ist ansteckend und so zieht er auch die Aufmerksamkeit eines kleinen Mädchens im Hintergrund auf sich. Sie schaut erstaunt und zugleich interessiert in seine Richtung.	92	7.38
Faces_114_h	Dein Bruder ist gerade Vater geworden und du stehst vor deinem neugeborenen Neffen. Er schaut dich direkt an und du erblickst seine großen, weit geöffneten Augen, seine niedliche Stupsnase und seinen kleinen Mund. Er scheint erstaunt und zugleich interessiert zu sein. Alles ist neu für ihn und er sieht dich zum ersten Mal. In seinem Gesichtsausdruck erkennst du große Neugierde. Mit seinem ersten Blick hat er dich bereits gefesselt. Du musst automatisch Lächeln und schaust weiterhin überglücklich in seine hervorstechenden Augen. Zu gern wüsstest du, was gerade in ihm vorgeht.	90	7.69
Faces_122_h	Neben dir im Stadtpark sitzt ein kleines Mädchen auf einer weißen Decke. Sie scheint nicht älter als 2 Jahre zu sein und ist zusammen mit ihrer Familie hier. Ihr lautes Lachen hat sofort deine Aufmerksamkeit geweckt. Enthusiastisch klatscht sie mit beiden Händen und weit geöffneten Mund. Ihre Augen sind leicht geschlossen und ihre Wangen hochgezogen. Ihr Lächeln ist ansteckend und sie strahlt vor purer Fröhlichkeit. So ein echtes und authentisches Lachen hast du lange nicht mehr gesehen! Etwas scheint sie sehr zu amüsieren und begeistern. Du genießt das Gefühl der Sorglosigkeit und Lebensfreude.	93	7.20

Faces_340_h	Du verbringst deinen freien Tag gemeinsam mit Freunden im örtlichen Schwimmbad. Die Sonne scheint und das Wasser ist angenehm warm. Du stehst gerade unter einem künstlichen Wasserfall und lässt das erfrischende Wasser auf dich herabfließen. Mit deinen Händen fährst du dir durch deine Haare. Deine Augen sind vollständig geschlossen und dein Mund ist leicht geöffnet. Du fühlst dich vollkommen entspannt und genießt den Moment in vollen Zügen. Deine Anspannung und Sorgen der letzten Tage scheinen in diesem Augenblick vergessen zu sein. Du spürst ein Gefühl der Sorglosigkeit und Ausgeglichenheit.	89	7.77
Objects_097_h	Es ist ein entspannter Sonntagmorgen. Du konntest ausschlafen und hast dir nun in aller Ruhe ein genussvolles Frühstück zubereitet. Du stellst alles auf deinen Holztisch in der Küche und setzt dich munter auf einen der Stühle. Du freust dich auf deinen morgendlichen Kaffee und betrachtest dein gesundes Frühstück: zwei Mehrkornbrotscheiben mit Käse, roten Tomaten und frischem Basilikum. Erst gestern hast du das Brot selbst gebacken. Der Kaffee ist noch heiß und du gießt Milch dazu. Nun kannst du unbeschwert dein Essen genießen und anschließend gestärkt in den Tag starten!	89	7.00
Objects_101_h	Du erwartest am Nachmittag noch Besuch von guten Freunden. Da euer letztes Treffen schon einige Wochen her ist, hast du dir überlegt, einen leckeren Kuchen zu backen. Du hast dir ein besonders anspruchsvolles Rezept herausgesucht und freust dich auf die Herausforderung. Nach 2 Stunden eifrigen Backens in der Küche ist dein Haselnusskuchen endlich fertig. Stolz betrachtest du dein Ergebnis: Er sieht genauso aus wie im Rezept. Der knusprig gebackene Teig, die cremige Nuss-Nougat-Schicht und als krönender Abschluss die zerkleinerten Haselnüsse oben drauf. Der verlockende Duft breitet sich bereits in der ganzen Küche aus.	93	7.56
People_055_h	Zusammen mit deinem kleinen Neffen unternimmst du gerade einen Spaziergang durch den nahegelegenen Wald. Er interessiert sich für deine Sonnenbrille und so borgst du ihm sie für einen kurzen Augenblick. Er hält sie sich verkehrt herum vor die Augen und beginnt über beide Ohren zu strahlen. Seine Mundwinkel sind nach oben gezogen	88	7.11

	und seine Wangen angehoben. Er scheint die Aufmerksamkeit voll und ganz zu genießen. Er zaubert dir ein Lächeln ins Gesicht und du erfreust dich an seinem positiven Gesichtsausdruck. Es ist ein Ausdruck der Fröhlichkeit und Begeisterung.		
People_067_h	Du verbringst deinen Traumurlaub auf den Philippinen. Gelassen schlenderst du durch die Straßen eines Küstenortes und bleibst vor einem großen, aus Holz geflochtenen Korb stehen. Er enthält zahlreiche Früchte und ist bis oben hin gefüllt. Ein Mann ist gerade dabei weitere Melonenscheiben klein zu schneiden. Interessiert betrachtest du all das frische Obst und Gemüse: Du siehst geschnittene Mango, erkennst eine Ananas, Gurken, mehrere kleine Kürbisse und Melonenstückchen. Auch reife Tomaten und Zucchini befinden sich unter dem Gemüse. Alles sieht sehr appetitlich aus und ist zum Verzehr fertig in kleine Schalen angerichtet.	91	6.80
People_176_h	Du bist bereits früh aufgestanden, um einen Spaziergang am Strand zu unternehmen. Zu deiner Überraschung entdeckst du am Wasser einen kleinen Jungen zusammen mit seinem Vater. Sie halten sich an den Händen und beobachten die zahlreichen Vögel im Wasser. Am Gesichtsausdruck des Vaters erkennst du vollkommene Freude und Zufriedenheit. Er scheint den gemeinsamen Augenblick zu genießen und sich daran zu erfreuen. Seine Mundwinkel sind nach oben gezogen und zu einem breiten Grinsen geformt. Sein Strahlen ist dir sofort aufgefallen. Du kannst die Vertrautheit zwischen beiden nahezu spüren.	87	7.31

Notes. Vignettes were constructed based on pictures from the *Nencki Affective Picture System* (NAPS; Marchewka et al., 2014).

[1] Picture ID refers to the corresponding ID from the NAPS.

[2] Further information regarding the online pilot studies can be requested from the 1st author.
