# Supplementary material for: From Abstract Symbols to Emotional (In-)Sights: An Eye Tracking Study on the Effects of Emotional Vignettes and Pictures
Source: Front Psychol. 2020 May 26;11:905. doi: 10.3389/fpsyg.2020.00905 (PMC7264705; doi:10.3389/fpsyg.2020.00905)
Supplement: Supplementary file 3 [file Table_3.pdf]

Supplementary Material

3 Emotionally neutral vignettes

Table S3

Emotionally neutral vignettes and their corresponding NAPS picture IDs

Picture ID[1]	Vignette	Number of words	Mean valence rating (1-9)[2]
Animals_031_h	In einer Zeitschrift über Insekten entdeckst du die Nahaufnahme einer Fliege. Sie befindet sich an einer roten Steinmauer. Durch das einfallende Licht kannst du ihren Schatten auf dem farbigen Hintergrund erkennen. Du siehst sämtliche Details: die feinen, durchsichtigen Flügel, die kleinen Fühler und Beine sowie die etwas größeren roten Augen. Der Körper ist schwarz mit einigen gelben Flächen durchsetzt. Sie sitzt vollkommen ruhig da. Die Flügel sind nicht weit ausgebreitet, sondern liegen eng am Körper an. Selten hast du eine Fliege so genau und nah betrachtet.	86	4.46
Objects_224_h	Du befindest dich auf einer Baustelle. Langsam näherst du dich einer der vielen Maschinen und schaust sie dir genauer an. Vor dir hängt ein großer Haken. Er sieht rostig aus und scheint schon viele Jahre in Benutzung zu sein. Dennoch wirkt er stabil und robust. Rechts von dem Haken erkennst du eine lange, dicke Metallkette. Auch sie ist rostig und gebraucht. Der Haken ist an ihr befestigt und hängt von oben herab. Du stellst dir vor, wie man mithilfe dieser Konstruktion schwere Materialien nach oben transportieren kann und sie die Arbeit erleichtert.	92	4.77

Landscapes_045_h	Du schlenderst durch eine kleine, ältere Gasse. Eine Reihe bemalter Türen erregt deine Aufmerksamkeit. Du richtest deinen Blick auf die auffälligen und hervorstechenden Türen. Sie sind hell- und dunkelblau angestrichen, scheinen aus Holz zu sein und sehen bereits etwas abgenutzt aus. An einigen Stellen ist die Farbe bereits abgesplittert. Vor den Türen befinden sich noch einige Treppenstufen. An einer Stelle scheint Wasser ausgeschüttet worden zu sein. Jede Tür ist mithilfe eines Metallschlosses verriegelt. Auch diese sehen etwas älter und benutzt aus. Weiter oben über den Türrahmen beginnt die gemauerte Fassade des Gebäudes.	92	5.31

Notes. Vignettes were constructed based on pictures from the *Nencki Affective Picture System* (NAPS; Marchewka et al., 2014).

[1] Picture ID refers to the corresponding ID from the NAPS.

[2] Further information regarding the online pilot studies can be requested from the 1st author.
