# Supplementary material for: From Abstract Symbols to Emotional (In-)Sights: An Eye Tracking Study on the Effects of Emotional Vignettes and Pictures
Source: Front Psychol. 2020 May 26;11:905. doi: 10.3389/fpsyg.2020.00905 (PMC7264705; doi:10.3389/fpsyg.2020.00905)
Supplement: Supplementary file 4 [file Table_4.pdf]

Supplementary Material

4 Linear Mixed-Effects Models: Evaluative Judgments

Intercepts-only models with by-subject and by-item random intercepts were computed using the lmer-function from the lme4 package (Bates et al., 2015b). Restricted maximum likelihood estimation was applied. Fixed-effects structures were obtained following a backward-elimination procedure (cf. Barr et al., 2013).

Initial models for the prediction of evaluative judgments (i.e., Valence Rating, Arousal Rating) possessed the following mathematical form:

$$Y_{si} = \beta_0 + S_{0s} + I_{0i} + \beta_1 X_1 + \beta_2 X_2 + \beta_3 X_3 + \beta_4 (X_1 X_2) + e_{si}, \tag{1}$$

where the subscripts s and i indicate subjects and items, respectively. Intercepts are denoted by the fixed-effects parameter β_0 and allowed to vary across subjects S_{0s} and items I_{0i} to account for both within-subjects and within-items variability. Hence, intercepts are adjusted for differences between subjects and items. Random effects and the error term e_{si} are assumed to be normally distributed with an expectation parameter of zero and an unknown variance parameter (cf. Barr et al., 2013):

$$\begin{aligned} S_{0s} &\sim N(0, \tau_{00}^2), \\ I_{0i} &\sim N(0, \omega_{00}^2), \\ e_{si} &\sim N(0, \sigma^2). \end{aligned}$$

The predictor variables X_1, X_2, and X_3 refer to Valence Category, Stimulus Domain, and Mood Rating, respectively. The interaction between Valence Category and Stimulus Domain is denoted by $X_1 X_2$ (afterward called Valence:Domain). Slope parameters (i.e., fixed-effects parameters) are denoted by β_1, β_2, β_3, and β_4. Two dependent variables (Y_{si}) were examined: Valence (Table S4) and Arousal Rating (Table S5).

4.1 Valence Rating

The following lmer specification corresponds to the initial model as denoted in Equation 1.

m_initial = lmer(sqrt(Valence Rating) ~ 1 + Valence Category[3] * Stimulus Domain[3] + Mood Rating[4] + (1|Subject) + (1|Item), data, REML=TRUE)

Table S4

Summary of the backward-elimination procedure for the prediction of valence ratings

	df_{Change}[1]	χ^2_{Change}[1]	log-likelihood[1]	χ^2	df	p-value[2]
Step 1			-435.34			
Intercept				11559.72	1	<.001
Valence Category[3]				1558.80	1	<.001
Stimulus Domain[3]				0.28	1	.59
Mood Rating[4]				0.11	1	.74
Valence:Domain				29.41	1	<.001
Step 2	1	0.10	-435.39			.75
Intercept				11638.91	1	<.001
Valence Category[3]				1558.73	1	<.001
Stimulus Domain[3]				0.25	1	.62
Valence:Domain				29.41	1	<.001

Notes. [1] Likelihood ratio tests were performed to compare the model fit of nested models differing in one degree of freedom (i.e., one parameter). Model fits are reported in terms of the log-likelihood and chi-squared distributed likelihood ratio test statistic. The anova-function from the stats package (R Core Team, 2019) was applied.

[2] Fixed effects were checked with Type III sum of squares statistics using the Anova-function from the car package (Fox and Weisberg, 2019).

[3] Effect coding was chosen for categorical variables.

[4] Metrical variables were centered prior to analysis to facilitate interpretations.
