# Supplementary material for: From Abstract Symbols to Emotional (In-)Sights: An Eye Tracking Study on the Effects of Emotional Vignettes and Pictures
Source: Front Psychol. 2020 May 26;11:905. doi: 10.3389/fpsyg.2020.00905 (PMC7264705; doi:10.3389/fpsyg.2020.00905)
Supplement: Supplementary file 5 [file Table_5.pdf]

Supplementary Material

4.2 Arousal Rating

The following lmer specification corresponds to the initial model (cf. Supplementary Material Table S4).

m_initial = lmer(sqrt(Arousal Rating) ~ 1 + Valence Category[3] * Stimulus Domain[3] + Mood Rating[4] + (1|Subject) + (1|Item), data, REML=TRUE)

Table S5

Summary of the backward-elimination procedure for the prediction of arousal ratings

	df_{Change}[1]	χ^2_{Change}[1]	log-likelihood[1]	χ^2	*df*	*p*-value[2]
Step 1			-1725.3			
Intercept				1951.65	1	<.001
Valence Category[3]				461.74	1	<.001
Stimulus Domain[3]				0.19	1	.67
Mood Rating[4]				1.37	1	.24
Valence:Domain				0.43	1	.51
Step 2	1	0.43	-1725.5			.51
Intercept				1951.87	1	<.001
Valence Category[3]				461.75	1	<.001
Stimulus Domain[3]				0.19	1	.66
Mood Rating[4]				1.37	1	.24
Step 3	1	0.19	-1725.6			.66
Intercept				1953.34	1	<.001
Valence Category[3]				461.74	1	<.001
Mood Rating[4]				1.25	1	.26
Step 4	1	1.24	-1726.2			.27
Intercept				1978.37	1	<.001
Valence Category				461.74	1	<.001

Notes. [1] Likelihood ratio tests were performed to compare the model fit of nested models differing in one degree of freedom (i.e. one parameter). Model fits are reported in terms of the log-likelihood and chi-squared distributed likelihood ratio test statistic. The anova-function from the stats package (R Core Team, 2019) was applied.

[2] Fixed effects were checked with Type III sum of squares statistics using the Anova-function from the car package (Fox and Weisberg, 2019).
[3] Effect coding was used for categorical variables.
[4] Metrical variables were centered prior to analysis to facilitate interpretations.
