# Supplementary material for: From Abstract Symbols to Emotional (In-)Sights: An Eye Tracking Study on the Effects of Emotional Vignettes and Pictures
Source: Front Psychol. 2020 May 26;11:905. doi: 10.3389/fpsyg.2020.00905 (PMC7264705; doi:10.3389/fpsyg.2020.00905)
Supplement: Supplementary file 6 [file Table_6.pdf]

Supplementary Material

5 Linear Mixed-Effects Models: Vignettes

Initial models for the prediction of eye movements in reading contained six predictors: Valence Rating (X_1), Arousal Rating (X_2), Mood Rating (X_3), Comprehensibility (X_4), Immersion Potential (X_5), and Emotion Induction Potential (X_6). The interaction between Valence and Arousal Rating was additionally included ($X_1 X_2$; afterward called Valence:Arousal). Three dependent variables (Y_{si}) were examined: Reading Speed (Table S6), mean First Fixation Duration (mean FFD; Table S7), and mean Total Reading Time (mean TRT; Table S8).

5.1 Reading Speed

The following lmer specification corresponds to the initial model.

m_initial = lmer(log(Reading Speed) ~ 1 + Valence Rating[3] * Arousal Rating[3] + Mood Rating[3] + Comprehensibility[3] + Immersion Potential[3] + Emotion Induction Potential[3] + (1|Subject) + (1|Item), data, REML=TRUE)

Table S6

Summary of the backward-elimination procedure for the prediction of Reading Speed

	df_{Change}[1]	χ^2_{Change}[1]	log-likelihood[1]	χ^2	*df*	*p*-value[2]
Step 1			753.13			
Intercept				18857.34	1	<.001
Valence Rating[3]				2.16	1	.14
Arousal Rating[3]				4.20	1	.04
Mood Rating[3]				0.59	1	.44
Comprehensibility[3]				0.02	1	.89
Immersion Potential[3]				4.08	1	.04
Emotion Induction Potential[3]				0.98	1	.32
Valence:Arousal				7.91	1	.005
Step 2	1	0.02	753.12			.88
Intercept				18881.58	1	<.001
Valence Rating[3]				2.22	1	.14
Arousal Rating[3]				4.25	1	.04
Mood Rating[3]				0.59	1	.44
Immersion Potential[3]				5.24	1	.02

Emotion Induction Potential[3]				0.99	1	.32
Valence:Arousal				7.90	1	.005
Step 3	1	0.61	752.81			.43
Intercept				19065.58	1	<.001
Valence Rating[3]				2.24	1	.13
Arousal Rating[3]				4.29	1	.04
Immersion Potential[3]				5.23	1	.02
Emotion Induction Potential[3]				0.99	1	.32
Valence:Arousal				7.97	1	.005
Step 4	1	1	752.31			.32
Intercept				19063.25	1	<.001
Valence Rating[3]				4.36	1	.04
Arousal Rating[3]				3.85	1	.05
Immersion Potential[3]				4.44	1	.04
Valence:Arousal				7.84	1	.005

Notes. [1] Likelihood ratio tests were performed to compare the model fit of nested models differing in one degree of freedom (i.e., one parameter). Model fits are reported in terms of the log-likelihood and chi-squared distributed likelihood ratio test statistic. The anova-function from the stats package (R Core Team, 2019) was applied.
[2] Fixed effects were checked with Type III sum of squares statistics using the Anova-function from the car package (Fox and Weisberg, 2019).
[3] Metrical variables were centered prior to analysis to facilitate interpretations.
