# Supplementary material for: From Abstract Symbols to Emotional (In-)Sights: An Eye Tracking Study on the Effects of Emotional Vignettes and Pictures
Source: Front Psychol. 2020 May 26;11:905. doi: 10.3389/fpsyg.2020.00905 (PMC7264705; doi:10.3389/fpsyg.2020.00905)
Supplement: Supplementary file 7 [file Table_7.pdf]

Supplementary Material

5.2 Mean FFD

The following lmer specification corresponds to the initial model.

m_initial = lmer(1/(mean FFD) ~ 1 + Valence Rating[3] * Arousal Rating[3] + Mood rating[3] + Comprehensibility[3] + Immersion Potential[3] + Emotion Induction Potential[3] + (1|Subject) + (1|Item), data, REML=TRUE)

Table S7

Summary of the backward-elimination procedure for the prediction of mean FFD

	df_{Change}[1]	χ^2_{Change}[1]	log-likelihood[1]	χ^2	*df*	*p*-value[2]
Step 1			10803			
Intercept				4095.96	1	<.001
Valence Rating[3]				0.01	1	.94
Arousal Rating[3]				0.43	1	.51
Mood Rating[3]				1.44	1	.23
Comprehensibility[3]				0.13	1	.72
Immersion Potential[3]				0.08	1	.78
Emotion Induction Potential[3]				0.53	1	.46
Valence:Arousal				0.60	1	.44
Step 2	1	0.08	10803			.77
Intercept				4098.58	1	<.001
Valence Rating[3]				0.03	1	.87
Arousal Rating[3]				0.44	1	.51
Mood Rating[3]				1.44	1	.23
Comprehensibility[3]				0.07	1	.80
Emotion Induction Potential[3]				0.47	1	.49
Valence:Arousal				0.62	1	.43
Step 3	1	0.07	10803			.79
Intercept				4101.45	1	<.001

Valence Rating[3]				0.01	1	.93
Arousal Rating[3]				0.49	1	.49
Mood Rating[3]				1.44	1	.23
Emotion Induction Potential[3]				0.60	1	.44
Valence:Arousal				0.62	1	.43
Step 4	1	0.63	10803			.43
Intercept				4100.92	1	<.001
Valence Rating[3]				0.14	1	.71
Arousal Rating[3]				0.34	1	.56
Mood Rating[3]				1.44	1	.23
Valence:Arousal				0.53	1	.46
Step 5	1	0.55	10802			.46
Intercept				4132.02	1	<.001
Valence Rating[3]				0.05	1	.83
Arousal Rating[3]				0.20	1	.65
Mood Rating[3]				1.47	1	.23
Step 6	1	0.05	10802			.82
Intercept				4130.06	1	<.001
Arousal Rating[3]				0.16	1	.69
Mood Rating[3]				1.47	1	.22
Step 7	1	0.16	10802			.69
Intercept				4124.06	1	<.001
Mood Rating[3]				1.48	1	.22
Step 8	1	1.52	10802			0.22
Intercept				4076.30	1	<.001

Notes.[1] Likelihood ratio tests were performed to compare the model fit of nested models differing in one degree of freedom (i.e., one parameter). Model fits are reported in terms of the log-likelihood and chi-squared distributed likelihood ratio test statistic. The anova-function from the stats package (R Core Team, 2019) was applied.
[2] Fixed effects were checked with Type III sum of squares statistics using the Anova-function from the car package (Fox and Weisberg, 2019).
[3] Metrical variables were centered prior to analysis to facilitate interpretations.
