# Supplementary material for: From Abstract Symbols to Emotional (In-)Sights: An Eye Tracking Study on the Effects of Emotional Vignettes and Pictures
Source: Front Psychol. 2020 May 26;11:905. doi: 10.3389/fpsyg.2020.00905 (PMC7264705; doi:10.3389/fpsyg.2020.00905)
Supplement: Supplementary file 8 [file Table_8.pdf]

Supplementary Material

5.3 Mean TRT

The following lmer specification corresponds to the initial model.

m_initial = lmer(sqrt(mean TRT) ~ 1 + Valence Rating[3] * Arousal Rating[3] + Mood Rating[3] + Comprehensibility[3] + Immersion Potential[3] + Emotion Induction Potential[3] + (1|Subject) + (1|Item), data, REML=TRUE)

Table S8

Summary of the backward-elimination procedure for the prediction of mean TRT

	df_{Change}[1]	χ^2_{Change}[1]	log-likelihood[1]	χ^2	*df*	*p*-value[2]
Step 1			6794.50			
Intercept				5790.55	1	<.001
Valence Rating[3]				3.29	1	.07
Arousal Rating[3]				0.86	1	.35
Mood Rating[3]				0.82	1	.37
Comprehensibility[3]				0.29	1	.59
Immersion Potential[3]				2.53	1	.11
Emotion Induction Potential[3]				0.43	1	.51
Valence:Arousal				2.24	1	.13
Step 2	1	0.32	6794.30			.57
Intercept				5797.94	1	<.001
Valence Rating[3]				3.13	1	.08
Arousal Rating[3]				0.92	1	.34
Mood Rating[3]				0.82	1	.36
Immersion Potential[3]				2.36	1	.12
Emotion Induction Potential[3]				0.47	1	.50
Valence:Arousal				2.19	1	.14
Step 3	1	0.48	6794.10			.49
Intercept				5800.35	1	<.001

Valence Rating[3]				5.11	1	.02
Arousal Rating[3]				0.76	1	.38
Mood Rating[3]				0.82	1	.37
Immersion Potential[3]				1.98	1	.16
Valence:Arousal				2.13	1	.14
Step 4	1	0.85	6793.60			.36
Intercept				5824.65	1	<.001
Valence Rating[3]				5.17	1	.02
Arousal Rating[3]				0.79	1	.38
Immersion Potential[3]				1.97	1	.16
Valence:Arousal				2.18	1	.14
Step 5	1	2.01	6792.60			.16
Intercept				5818.91	1	<.001
Valence Rating[3]				7.44	1	.01
Arousal Rating[3]				0.77	1	.38
Valence:Arousal				2.39	1	.12
Step 6	1	2.39	6791.40			.12
Intercept				5872.66	1	<.001
Valence Rating[3]				6.02	1	.01
Arousal Rating[3]				0.34	1	.56
Step 7	1	0.35	6791.30			.55
Intercept				5850.93	1	<.001
Valence Rating[3]				6.05	1	.01

Notes. [1] Likelihood ratio tests were performed to compare the model fit of nested models differing in one degree of freedom (i.e., one parameter). Model fits are reported in terms of the log-likelihood and chi-squared distributed likelihood ratio test statistic. The anova-function from the stats package (R Core Team, 2019) was applied.
[2] Fixed effects were checked with Type III sum of squares statistics using the Anova-function from the car package (Fox and Weisberg, 2019).
[3] Metrical variables were centered prior to analysis to facilitate interpretations.
