# Supplementary material for: From Abstract Symbols to Emotional (In-)Sights: An Eye Tracking Study on the Effects of Emotional Vignettes and Pictures
Source: Front Psychol. 2020 May 26;11:905. doi: 10.3389/fpsyg.2020.00905 (PMC7264705; doi:10.3389/fpsyg.2020.00905)
Supplement: Supplementary file 9 [file Table_9.pdf]

Supplementary Material

6 Linear Mixed-Effects Models: Pictures

Initial models for the prediction of eye movements in picture viewing contained three predictor variables (X_1: Valence Rating; X_2: Arousal Rating; X_3: Mood Rating) and the interaction between Valence and Arousal Rating (X_1X_2; afterward called Valence:Arousal). Three dependent variables (Y_{si}) were examined: Mean Saccade Amplitude (Table S9), Total Number of Fixations (Table S10), and Mean Fixation Duration (Table S11).

6.1 Mean Saccade Amplitude

The following lmer specification corresponds to the initial model.

m_initial = lmer(sqrt(Mean Saccade Amplitude) ~ 1 + Valence Rating[3] * Arousal Rating[3] + Mood Rating[3] + (1|Subject) + (1|Item), data, REML=TRUE)

Table S9

Summary of the backward-elimination procedure for the prediction of Mean Saccade Amplitude

	df_{Change}[1]	χ^2_{Change}[1]	log-likelihood[1]	χ^2	*df*	*p*-value[2]
Step 1			359.23			
Intercept				4730.52	1	<.001
Valence Rating[3]				0.39	1	.53
Arousal Rating[3]				0.78	1	.38
Mood Rating[3]				0.34	1	.56
Valence:Arousal				3.54	1	.06
Step 2	1	0.35	359.05			.56
Intercept				4750.77	1	<.001
Valence Rating[3]				0.37	1	.54
Arousal Rating[3]				0.76	1	.38
Valence:Arousal				3.47	1	.06
Step 3	1	3.47	357.32			.06
Intercept				4865.81	1	<.001
Valence Rating[3]				0.06	1	.80
Arousal Rating[3]				0.39	1	.53

Step 4	1	0.05	357.30			.82
Intercept				4915.04	1	<.001
Arousal Rating[3]				0.34	1	.56
Step 5	1	0.34	357.12			.56
Intercept				4871.80	1	<.001

Notes. [1] Likelihood ratio tests were performed to compare the model fit of nested models differing in one degree of freedom (i.e., one parameter). Model fits are reported in terms of the log-likelihood and chi-squared distributed likelihood ratio test statistic. The anova-function from the stats package (R Core Team, 2019) was applied.
[2] Fixed effects were checked with Type III sum of squares statistics using the Anova-function from the car package (Fox and Weisberg, 2019).
[3] Metrical variables were centered prior to analysis to facilitate interpretations.
