# Supplementary material for: From Abstract Symbols to Emotional (In-)Sights: An Eye Tracking Study on the Effects of Emotional Vignettes and Pictures
Source: Front Psychol. 2020 May 26;11:905. doi: 10.3389/fpsyg.2020.00905 (PMC7264705; doi:10.3389/fpsyg.2020.00905)
Supplement: Supplementary file 10 [file Table_10.pdf]

Supplementary Material

6.2 Total Number of Fixations

The following lmer specification corresponds to the initial model.

m_initial = lmer((Total Number of Fixations)^2 ~ 1 + Valence Rating[3] * Arousal Rating[3] + Mood Rating[3] + (1|Subject) + (1|Item), data, REML=TRUE)

Table S10

Summary of the backward-elimination procedure for the prediction of Total Number of Fixations

	df_{Change}[1]	χ^2_{Change}[1]	log-likelihood[1]	χ^2	*df*	*p*-value[2]
Step 1			-7937.00			
Intercept				665.60	1	<.001
Valence Rating[3]				0.00	1	.996
Arousal Rating[3]				1.78	1	.18
Mood Rating[3]				0.57	1	.45
Valence:Arousal				2.02	1	.15
Step 2	1	0.59	-7937.30			.44
Intercept				670.49	1	<.001
Valence Rating[3]				0.0001	1	.99
Arousal Rating[3]				1.81	1	.18
Valence:Arousal				1.97	1	.16
Step 3	1	1.98	-7938.30			.16
Intercept				672.03	1	<.001
Valence Rating[3]				0.10	1	.76
Arousal Rating[3]				2.49	1	.11
Step 4	1	0.09	-7938.30			.76
Intercept				647.81	1	<.001
Arousal Rating[3]				2.47	1	.12
Step 5	1	2.46	-7939.50			.12
Intercept				661.78	1	<.001

Notes. [1] Likelihood ratio tests were performed to compare the model fit of nested models differing in one degree of freedom (i.e., one parameter). Model fits are reported in terms of the log-likelihood and chi-squared distributed likelihood ratio test statistic. The anova-function from the stats package (R Core Team, 2019) was applied.
[2] Fixed effects were checked with Type III sum of squares statistics using the Anova-function from the car package (Fox and Weisberg, 2019).
[3] Metrical variables were centered prior to analysis to facilitate interpretations.
