# Supplementary material for: From Abstract Symbols to Emotional (In-)Sights: An Eye Tracking Study on the Effects of Emotional Vignettes and Pictures
Source: Front Psychol. 2020 May 26;11:905. doi: 10.3389/fpsyg.2020.00905 (PMC7264705; doi:10.3389/fpsyg.2020.00905)
Supplement: Supplementary file 11 [file Table_11.pdf]

Supplementary Material

6.3 Mean Fixation Duration

The following lmer specification corresponds to the initial model.

m_initial = lmer(1/(Mean Fixation Duration) ~ 1 + Valence Rating[3] * Arousal Rating[3] + Mood Rating[3] + (1|Subject) + (1|Item), data, REML=TRUE)

Table S11

Summary of the backward-elimination procedure for the prediction of Mean Fixation Duration

	df_{Change}[1]	χ^2_{Change}[1]	log-likelihood[1]	χ^2	*df*	*p*-value[2]
Step 1			9828.10			
Intercept				1858.19	1	<.001
Valence Rating[3]				0.01	1	.91
Arousal Rating[3]				0.52	1	.47
Mood Rating[3]				0.99	1	.32
Valence:Arousal				2.37	1	.12
Step 2	1	1.02	9827.60			.31
Intercept				1858.55	1	<.001
Valence Rating[3]				0.02	1	.90
Arousal Rating[3]				0.54	1	.46
Valence:Arousal				2.29	1	.13
Step 3	1	2.30	9826.50			.13
Intercept				1878.91	1	<.001
Valence Rating[3]				0.19	1	.66
Arousal Rating[3]				0.96	1	.33
Step 4	1	0.18	9826.40			.67
Intercept				1889.70	1	<.001
Arousal Rating[3]				0.80	1	.37
Step 5	1	0.80	9826.00			.37
Intercept				1866.80	1	<.001

Notes. [1] Likelihood ratio tests were performed to compare the model fit of nested models differing in one degree of freedom (i.e., one parameter). Model fits are reported in terms of the log-likelihood and chi-squared distributed likelihood ratio test statistic. The anova-function from the stats package (R Core Team, 2019) was applied.
[2] Fixed effects were checked with Type III sum of squares statistics using the Anova-function from the car package (Fox and Weisberg, 2019).
[3] Metrical variables were centered prior to analysis to facilitate interpretations.
